# Biocontrol potential and molecular basis of predation in a marine raptorial ciliate

**DOI:** 10.1093/ismejo/wrag053

**Published:** 2026-03-13

**Authors:** Jiao Pan, Jiahao Ni, Yaohai Wang, Ziguang Deng, Hongwei Yue, Kangqiao Dong, Yichen Li, Zhongze Lei, Ziming Ma, Gongze Hu, Runda Chi, Zhongyu Chang, Qikai Chen, Yujun Cai, Hanlin Shen, Runzhi Shi, Wei Yang, Xinpeng Fan, Weiyi Li, Zhiqiang Ye, Michael Lynch, Yu Zhang, Hongan Long

**Affiliations:** Key Laboratory of Evolution and Marine Biodiversity (Ministry of Education), Institute of Evolution and Marine Biodiversity, Ocean University of China, Qingdao, Shandong Province 266003, China; Laboratory for Marine Biology and Biotechnology, Qingdao Marine Science and Technology Center, Qingdao, Shandong Province 266237, China; Key Laboratory of Evolution and Marine Biodiversity (Ministry of Education), Institute of Evolution and Marine Biodiversity, Ocean University of China, Qingdao, Shandong Province 266003, China; Key Laboratory of Evolution and Marine Biodiversity (Ministry of Education), Institute of Evolution and Marine Biodiversity, Ocean University of China, Qingdao, Shandong Province 266003, China; Key Laboratory of Evolution and Marine Biodiversity (Ministry of Education), Institute of Evolution and Marine Biodiversity, Ocean University of China, Qingdao, Shandong Province 266003, China; Key Laboratory of Evolution and Marine Biodiversity (Ministry of Education), Institute of Evolution and Marine Biodiversity, Ocean University of China, Qingdao, Shandong Province 266003, China; School of Life Sciences, East China Normal University, Shanghai 200241, China; Key Laboratory of Evolution and Marine Biodiversity (Ministry of Education), Institute of Evolution and Marine Biodiversity, Ocean University of China, Qingdao, Shandong Province 266003, China; Key Laboratory of Evolution and Marine Biodiversity (Ministry of Education), Institute of Evolution and Marine Biodiversity, Ocean University of China, Qingdao, Shandong Province 266003, China; Key Laboratory of Evolution and Marine Biodiversity (Ministry of Education), Institute of Evolution and Marine Biodiversity, Ocean University of China, Qingdao, Shandong Province 266003, China; Key Laboratory of Evolution and Marine Biodiversity (Ministry of Education), Institute of Evolution and Marine Biodiversity, Ocean University of China, Qingdao, Shandong Province 266003, China; Key Laboratory of Evolution and Marine Biodiversity (Ministry of Education), Institute of Evolution and Marine Biodiversity, Ocean University of China, Qingdao, Shandong Province 266003, China; Key Laboratory of Evolution and Marine Biodiversity (Ministry of Education), Institute of Evolution and Marine Biodiversity, Ocean University of China, Qingdao, Shandong Province 266003, China; Key Laboratory of Evolution and Marine Biodiversity (Ministry of Education), Institute of Evolution and Marine Biodiversity, Ocean University of China, Qingdao, Shandong Province 266003, China; Key Laboratory of Evolution and Marine Biodiversity (Ministry of Education), Institute of Evolution and Marine Biodiversity, Ocean University of China, Qingdao, Shandong Province 266003, China; Key Laboratory of Evolution and Marine Biodiversity (Ministry of Education), Institute of Evolution and Marine Biodiversity, Ocean University of China, Qingdao, Shandong Province 266003, China; Key Laboratory of Evolution and Marine Biodiversity (Ministry of Education), Institute of Evolution and Marine Biodiversity, Ocean University of China, Qingdao, Shandong Province 266003, China; Key Laboratory of Evolution and Marine Biodiversity (Ministry of Education), Institute of Evolution and Marine Biodiversity, Ocean University of China, Qingdao, Shandong Province 266003, China; School of Life Sciences, East China Normal University, Shanghai 200241, China; Department of Genetics, Stanford University School of Medicine, Stanford CA 94305, United States; School of Life Sciences, Central China Normal University, Wuhan, Hubei Province 430079, China; Biodesign Center for Mechanisms of Evolution, Arizona State University, Tempe AZ 85287, United States; School of Mathematics Science, Ocean University of China, Qingdao, Shandong Province 266100, China; Key Laboratory of Evolution and Marine Biodiversity (Ministry of Education), Institute of Evolution and Marine Biodiversity, Ocean University of China, Qingdao, Shandong Province 266003, China; Laboratory for Marine Biology and Biotechnology, Qingdao Marine Science and Technology Center, Qingdao, Shandong Province 266237, China

**Keywords:** population dynamics, experimental ecology, functional genomics, predation, protozoa

## Abstract

Predator–prey interactions are widespread across organisms and are key drivers of morphological and behavioral evolution. Despite this, predation remains poorly understood among microbial eukaryotes, mostly due to the absence of a tractable experimental system that allows quantitative, reproducible investigation. This study establishes the marine raptorial ciliate *Chaenea vorax* as a highly efficient predator, with Rosenzweig–MacArthur model simulations based on predation data showing that only a few dozen individuals can eliminate the vast majority of the facultatively pathogenic ciliate *Uronema marinum* within 1–2 days, providing a quantitative basis for developing predator-based biocontrol strategies in aquaculture. Genomic analysis shows that *C. vorax* possesses a highly fragmented macronuclear genome enriched with predation-related pathways, including calcium-mediated contractility, cellular proteolysis, toxin expulsion systems, among others. Transcriptomic profiling during predation events further demonstrates significant upregulation of genes involved in cytoskeletal remodeling, proteolytic activity, and cellular detoxification. Evolutionary analyses suggest that *C. vorax* has an extremely long evolutionary history, exceptionally high nucleotide diversity even among ciliates, and gene family expansions linked to predatory adaptation. Although the prey possesses certain defensive mechanisms (e.g. trichocysts), these are largely ineffective against short-term predation in closed aquatic environments. These findings provide fundamental insights into the molecular basis of predation in ciliates and suggest the potential utility of *C. vorax* in biocontrol applications targeting pathogenic ciliates.

## Introduction

Predation is a fundamental process driving material transfer and energy flow within microbial food webs, serving as a key mechanism for population control and resource allocation in aquatic ecosystems [1, 2]. Within this framework, ciliates play a crucial functional role, and some of them act as major predators exerting top-down control on microbial community structure and facilitating carbon turnover [3–8]. Whereas ciliate predation on bacteria, microalgae, and other non-ciliate groups has been extensively documented [9–12], and ciliates themselves are known to be preyed upon by various organisms such as copepods, nematodes, turbellarians, and flagellates [13–17], predation among ciliate species—especially in marine systems—remains poorly explored. Existing studies have largely focused on freshwater models, such as *Didinium* preying on *Paramecium* or *Colpoda* [3, 5, 6], leaving a significant knowledge gap regarding the mechanisms and ecological impacts of marine intra-Ciliophora predation. The stark differences in ionic strength and viscosity between freshwater and marine habitats suggest that predation mechanisms may not be directly comparable, necessitating the establishment of a dedicated marine model to uncover environment-specific adaptations [18, 19]. Furthermore, understanding predation among ciliates at different trophic levels is essential for quantifying their crucial role within the microbial loop. Their specific predatory interactions, including size-selective feeding and prey switching, can reshape microbial community composition and alter the fate of organic matter. Therefore, elucidating the molecular and behavioral basis of predation between ciliates is not only a question of mechanistic biology but also central to modeling energy flow and carbon cycling in ecosystems.

Recent advances have begun to uncover the molecular basis of predation in ciliates. For instance, haptorian ciliates have evolved predation-related gene families involved in membrane transport, hydrolytic activity, and toxin production [20]. In the Litostomatea class, gene expansions in ion channels and metabolic pathways support their predatory behaviors [21], whereas ultra-rapid prey capture mechanisms have been associated with unique microtubular architectures, as demonstrated in *Lacrymaria olor* [22, 23]. Despite these insights, current research remains predominantly focused on predator adaptations, with limited emphasis on the predation process—from prey detection and capture to digestion—especially in marine ciliates, particularly at the molecular level.

In this study, we isolated and established two marine ciliates with the predator–prey relationship: *Chaenea vorax* PJ13002, a raptorial ciliate, and *Uronema marinum* PJ20101A, which is preyed upon by the former. *Uronema marinum* is a dominant ciliate species in coastal waters, as well as a facultative pathogen in aquaculture, causing primary or secondary infections that can be lethal to fish, shrimp, and shellfish [24–27]. Intensive farming practices, characterized by crowding and eutrophication, often trigger proliferations of such ciliates, leading to substantial economic losses. Current control methods for pathogenic ciliates rely heavily on physical and chemical treatments [28], which are costly, labor-intensive, and pose environmental and food safety risks.

We first quantified and modeled the predation efficiency and biocontrol potential of *C. vorax* against *U. marinum*, followed by multi-omics and evolutionary analyses to uncover the molecular basis of its predatory behavior. Specifically, to uncover the genomic basis of adaptation, we conducted genome assembly, gene prediction, and pathway analysis of the predator. Meanwhile, to dynamically capture the molecular mechanisms underlying prey digestion and other predation stages, we performed differential gene expression analyses, including low-input RNAseq to resolve cellular-level responses. Furthermore, evolutionary analyses were conducted to understand the genetic constraints and pressures on both predator and prey. Unlike previous studies centered on coevolutionary arms races or defensive strategies, this study focuses on the predation process while establishing a laboratory model that not only addresses fundamental questions in evolutionary ecology but also offers translational potential for sustainable biocontrol strategies against facultative pathogenic ciliates.

## Materials and methods

### Isolation, culture, and identification of species

*Uronema marinum* PJ20101A (36°3′31″N 120°19′46″E; 8.0°C; pH = 8.00; 01 December 2017), *Chaenea vorax* PJ13002 (36°3′31″N 120°20′6″E; 27.0°C; pH = 8.06; 13 July 2019) and *Chaenea vorax* C13 (36°5′40″N 120°28′33″E; 21.3°C; pH = 7.50; 16 July 2023) were all collected from the coastal water of Qingdao, Shandong Province, China. Each strain was established from one single cell, which was rinsed by serial dilution in autoclaved seawater with 10 μg/ml Penicillin-Streptomycin-Amphotericin B (Cat. No.: 03-033-1B; Biological Industries). Then we cultured *U. marinum* PJ20101A with the food bacteria *Escherichia coli* K12 MG1655 (prepared in autoclaved seawater, OD_600_ ~ 0.3) at 25°C. The strains of *C. vorax* were inoculated into and maintained in the *U. marinum* culture.

The living cells were randomly chosen and placed on a slide and observed using bright field and differential interference contrast microscopy at 1000× magnification (Nikon Eclipse Ni-U). To reveal the nuclear apparatus, the cells were stained with Hoechst 33342 solution at a 1:9 (vol/vol) dilution for one hour and then observed using the fluorescent module of the microscope. The ultrastructure of trichocysts in *U. marinum* was examined by transmission electron microscopy (TEM). Following a protocol [29], samples were sectioned using a Leica EM UC7 ultramicrotome, stained with uranyl acetate and lead citrate, and imaged under a Hitachi HT7700 TEM at an operating voltage of 80 kV. We identified species by morphological features and 18S rRNA gene sequences. Genomic DNA was obtained by directly lysing ~10 ciliate cells using RoomTemp Sample Lysis Kit (Vazyme, P073-01). The 18S rRNA gene was amplified using primers EukA (5′-AACCTGGTTGATCCTGCCAGT-3′) and EukB (5′-TGATCCTTCTGCAGGTTCACCTAC-3′) in a PCR system consisting of 1 μL lysed ciliates, 7.5 μL 2× Phanta Flash Master Mix (Vazyme, P501-01), 0.5 μL forward primer, 0.5 μL reverse primer, and 5.5 μL sterilized water. A touchdown PCR protocol was done with the following conditions: initial denaturation at 98°C for 30 s; followed by 18 cycles of denaturation at 98°C for 30 s, annealing for 30 s (starting at 69°C and decreasing by 1°C per cycle to 51°C), and extension at 72°C for 30 s; then another 18 cycles of denaturation at 98°C for 30 s, annealing at 51°C for 30 s, and extension at 72°C for 30 s; with a final extension at 72°C for 5 min. PCR products were purified and subjected to bidirectional Sanger sequencing on a ABI 3730XL sequencer of Tsingke Biotechnology Co., Ltd., Beijing.

### Predation experiment

Six sterile 100 ml conical flasks were prepared, each containing 40 ml of bacterial suspension (OD_600_ ≈ 0.3) and ~4000 *U. marinum* PJ20101A cells. Cultures were maintained at 25°C for 48 h. Subsequently, three randomly selected flasks were treated with 50 *C. vorax* PJ13002 cells, while the remaining three served as controls. All flasks were then incubated at 18°C. Then samples were taken every 24 h after thorough shaking, and 400 μL culture was taken and fixed with 100 μL Bouin’s solution. 100 μL of the evenly mixed and fixed culture was counted using a plankton counter (Gridded Sedgewick Rafter, 1mm^2^) for the number of cells (N), under a dissecting microscope. The density in each flask was calculated by 12.5 × N cells/ml, where N is the number of cells counted under the microscope. The experiment continued until the population of *U. marinum* PJ20101A stabilized, then the growth curves of prey in the treatment and the control were finally plotted.

### Mathematical modeling and parameter estimation of predator–prey dynamics

To quantitatively analyze the predator–prey dynamics between *C. vorax* PJ13002 (predator) and *U. marinum* PJ20101A (prey), we implemented a two-step ordinary differential equation (ODE) modeling framework. Prey growth in the absence of predators was modeled independently before constructing the full predator–prey system. For the prey without predator cultures, trajectories were first fitted with a logistic growth model,


1 \begin{eqnarray*} \frac{dN}{dt}= rN\left(1-\frac{N}{K}\right) \end{eqnarray*}

where $N$ denotes prey density (cells/ml), $r$ is the intrinsic growth rate (day^−1^), and $K$ is the carrying capacity (cells/ml). To ensure computational efficiency and numerical stability, the parameters $r$ and $K$ were estimated by nonlinear least-squares fitting using the Levenberg–Marquardt algorithm implemented in scipy.optimize.curve_fit (Python 3.11, SciPy v1.10), with initial guesses based on the observed magnitude of the time-series data. Fit quality was assessed by the coefficient of determination $\left({R}^2\right)$. Although the logistic model adequately captured early exponential growth, it systematically underestimated late-stage declines. To account for this, we introduced a bi-phasic extension in which the logistic solution ${N}_{logistic}(t)$ was multiplied by an exponential decay factor,


2 \begin{eqnarray*} {N}_{bi- phasic}(t)={N}_{logistic}(t){e}^{-k\times \mathit{\max}\left(t-{t}_{s,}0\right)} \end{eqnarray*}

where ${t}_s$ is the switch time of density-independent mortality, and $k$ is the decay rate per day.

The predator–prey interaction was modeled by the Rosenzweig–MacArthur differential system. To identify an appropriate functional response $\Big(\frac{a{N}^q}{1+ ah{N}^q}\Big)$, we compared Holling type II ($q=1$) and type III ($q=2$) formulations, and further fitted a generalized Hill-type functional response with a free exponent $q$ (Supplementary Table S1). The intrinsic prey growth parameters ($r$ and $K$) were fixed at values estimated from prey-alone dynamics. For each candidate formulation, the coupled ODE system was numerically integrated and fitted simultaneously to prey and predator time-series by minimizing the joint residual sum of squares between observed and simulated trajectories. All models were optimized under identical initialization and convergence settings to ensure comparability. Model selection was based on Akaike Information Criterion (AIC) and Bayesian Information Criterion (BIC) calculated from the joint residual sum of squares under a Gaussian error assumption. Although the type II model yielded slightly lower AIC and BIC values, the differences relative to the type III model were small (ΔAIC <1; ΔBIC <1) and therefore not decisive. Moreover, the type II model failed to reproduce predator dynamics, whereas both the type III and Hill-type models provided an excellent fit to predator time series. In the generalized Hill formulation, the estimated exponent converged to values close to 2. We therefore adopted the Holling type III functional response as the most parsimonious model that adequately captures the coupled predator–prey dynamics [30].


3 \begin{eqnarray*} \frac{dN}{dt}= rN\left(1-\frac{N}{K}\right)-\frac{a{N}^2}{1+ ah{N}^2}P \end{eqnarray*}

4 \begin{eqnarray*} \frac{dP}{dt}=e\frac{a{N}^2}{1+ ah{N}^2}P-\beta P \end{eqnarray*}

where $P(t)$ denotes predator density (cells/ml), $a$ is the attack rate (ml predator^−1^ day^−1^), $h$ the handling time (day), $e$ the conversion efficiency, and $\beta$ the predator death (day^−1^). The quadratic prey term in the functional response introduces a sigmoidal curve, reflecting reduced predation efficiency at low prey densities due to prey refuge or predator learning effects.

Numerical integration of the system was performed with the odeint integrator in SciPy under strict tolerances (mxstep = 100 000; atol = 10^−6^; rtol = 10^−6^). Parameters $a,h,e,\beta$ were estimated by minimizing the joint residual sum of squares between observed and simulated trajectories of prey and predator, with optimization carried out using the Nelder–Mead simplex algorithm (lmfit.minimize, method = “nelder”).

Parameter uncertainty was quantified using nonparametric bootstrap resampling (500 iterations). In each replicate, resampled time-series were re-fitted under the same Nelder–Mead routine, and only biologically plausible parameter sets were retained. Confidence intervals for estimated $\hat{a}$, $\hat{h}$, $\hat{e}$, $\hat{\beta}$ were derived from log-transformed percentile estimates of bootstrap distributions obtained from model fitting, and geometric means were reported as bias-corrected central estimates. Intrinsic growth rate of *C. vorax* was calculated by ${\hat{r}}_{P,\mathit{\max}}=\frac{\hat{e}}{\hat{h}}-\hat{\beta}$, derived from equation (4) when prey density is large and sufficient for predation.

### Predator-based control threshold analysis

The fitted predator–prey system was further applied to simulate control scenarios to quantify the minimal predator abundance required for prey suppression. Let $N(t)$ and $P(t)$ denote prey and predator densities at time $t$, respectively, with initial conditions $N(0)={N}_0$ and $P(0)={P}_0$. For a given specified prey suppression threshold ${N}^{\ast }$ and finite time $T$, the control objective was defined as


\begin{equation*}N(t)<{N}^{\ast}\mathrm{for}\ \mathrm{some}\ t\le T. \end{equation*}

To determine the minimal predator density satisfying this objective, we systematically varied the initial predator abundance ${P}_0$ while keeping all other parameters fixed at their fitted values. The minimal (infimum of set) effective predator density was then defined as


\begin{equation*}{P}_0^{min}=\operatorname{inf}\left({P}_0|\exists t\le T\ \mathrm{such}\ \mathrm{that}\ N(t)<{N}^{\ast}\right). \end{equation*}

In practice, numerical computation approximated this criterion by evaluating the terminal prey density $N(T).$ To compute ${P}_0^{min}$, we implemented a binary search routine. For each candidate ${P}_0$, the fitted predator–prey system was numerically integrated over the interval [$0,T]$ using the same ODE solver (odeint). At each iteration, the terminal prey density $N(T)$ was compared with the threshold ${N}^{\ast }$. If $N(T)<{N}^{\ast }$, the candidate ${P}_0$ was deemed sufficient and the upper bound of the search interval was reduced; otherwise, the lower bound was raised. Iterations continued until convergence within a predefined tolerance $({10}^{-3})$ or until the maximum iteration limit (100) was reached. The resulting ${P}_0^{min}$ was thus the smallest initial predator abundance capable of reducing prey density below the target threshold ${N}^{\ast }$ within the given time $T$.

### DNA/RNA extraction and sequencing for *Uronema marinum* PJ20101A and *Chaenea vorax* PJ13002/C13

To extract genomic DNA for long-read sequencing, *U. marinum* PJ20101A were cultured in 400 ml of bacterial suspension (OD_600_ ~ 0.3) in 1 l flasks for 4 days. 300 ml of the upper-layer culture was then centrifuged at 1500 g for 5 min to enrich cells. A sucrose-gradient protocol was applied to reduce bacterial contamination. Briefly, the enriched cells were then rinsed to remove any residual medium using a solution composed of 10 mM Tris (pH 7.2), 0.25 M sucrose, and 2 mM MgCl_2_, followed by centrifugation at 1500 g for 2 min in a Sigma 3-18KS centrifuge with a swing out rotor. Subsequently, the cells were lysed with a lysis solution composed of 10 mM Tris (pH 7.2), 0.25 M sucrose, 2 mM MgCl_2_, 0.5% Nonidet P40, and 0.1% sodium deoxycholate. The lysis mixture was then centrifuged at 1500 g for 2 min. The supernatant was discarded, and the lysis solution was added again. Following a 30-min incubation on ice, the mixture was centrifuged at 1500 g for 2 min, which yielded white precipitates of macronuclei. The macronuclear genomic DNA was extracted using the MasterPure Complete DNA & RNA Purification Kit (Cat. No. MC85200; Lucigen, USA). To extract RNA for structural annotation of the macronuclear genome, *U. marinum* PJ20101A was cultured using the same conditions as for DNA extraction. Total RNA was extracted using the MasterPure Complete DNA & RNA Purification Kit after 2 days (active predation, with frequent prey captures observed) and 4 days (non-predation, referring specifically to the post-predation state when prey is largely depleted and active feeding has ceased, not a condition where prey was never present) of culture, respectively to capture RNA transcribed in various life stages. We then constructed RNA sequencing libraries using the NEBNext Single Cell/Low Input RNA Library Prep Kit for Illumina (NEB, Cat. No.: E6420S).

To extract nucleic acids of *C. vorax* for genome assembly and annotation, 1000 *U. marinum* PJ20101A were first added to 150 × 25 mm petri dish containing 100 ml bacterial suspension (OD_600_ ~ 0.3) and cultured for 2 days. Then, another 100 *C. vorax* PJ13002 were added and cultured for about 4 days. After *U. marinum* PJ20101A became rare, *C. vorax* cells were pipetted and transferred into a 50 ml conical tube, and centrifuged at 1000 g for 5 min. The MasterPure kit was then used to extract genomic DNA and total RNA.

To determine gene expression in predation and non-predation states of *C. vorax* C13, we picked three *C. vorax* C13 cells in predation and another three in non-predation conditions to construct the low-input RNAseq libraries with four replicates in each state. The Single Cell Full Length mRNA Amplification Kit (Vazyme, Cat. No.: N712) and the TruePrep DNA Library Prep Kit V2 for Illumina (Vazyme, Cat. No.: TD503) were used for mRNA reverse transcription, resulting in the construction of six successful RNA libraries.

To assemble *U. marinum* PJ20101A, we generated HiFi reads using Circular Consensus Sequencing with a PacBio Sequel II sequencer (Berry Genomics, Beijing, China). For *C. vorax* PJ13002, we prepared the library using the ligation sequencing kit (SQK-LSK109), followed by long-read sequencing on an Oxford Nanopore PromethION. Macronuclear genomic DNA libraries of *U. marinum* PJ20101A, *C. vorax* PJ13002, and *C. vorax* C13 were prepared by the TruSeq Nano kit (Cat. No. 20015964; Illumina, USA), and sequenced on the NovaSeq 6000 PE150 platform (Illumina) at Novogene (Beijing, China). Additionally, the NovaSeq 6000 PE150 sequencing was also performed for all of the above RNA libraries.

### Macronuclear genome assembly and annotation

We obtained 10 Gbp PacBio HiFi long clean reads and 5 Gbp short clean reads of *U. marinum* PJ20101A, and 19 Gbp Nanopore long clean reads and 20 Gbp short clean reads of *C. vorax* PJ13002, after removing sequences that can be aligned to the genome of *Escherichia coli* K12 MG1655 (GenBank: GCA_000005845.2). The pbccs (https://github.com/nlhepler/pbccs), extracthifi v1.0.0 (https://github.com/nlhepler/pbccs), and SAMtools v0.1.9 [31] were used to convert raw subreads into HiFi reads. For Nanopore raw reads of *C. vorax* PJ13002, we used Guppy v4.5.4 (Oxford Nanopore Technologies) for base calling [32], and filtered out reads with the base quality score < 8 and length < 1000 bp using NanoFilt v2.7.1 [33]. Next, the high-quality reads were aligned to the reference genome of the food bacteria using Minimap2 (-x map-ont) with the default mode, and the sequences that could be aligned were removed [34]. For Illumina sequences, fastp v0.20.1 was used to trim adaptors and filter the low-quality reads with the parameter: “-u 20 -q 20” [35]. The resulting reads were aligned to the food bacteria reference genome using BWA v0.7.17 with default setting [36] and unaligned data was extracted using SAMtools v0.1.9 with -bf 12.

The draft genomes were assembled by IPA HiFi Genome Assembler v1.8.0 (https://github.com/PacificBiosciences/pbbioconda) and SPAdes v2.2 [37] for *U. marinum* PJ20101A and *C. vorax* PJ13002, respectively. Then three rounds of polishing with Racon v1.4.3 [38] and another three additional rounds with Pilon v1.24 [39] for the draft genomes were performed. Contigs with GC content higher than 25% and 55% were removed for *U. marinum* PJ20101A and *C. vorax* PJ13002 respectively, based on GC-content-distribution peaks of contigs to exclude bacterial contamination. Contigs shorter than 300 bp in length were removed. Contigs were queried against the bacterial genome database using BLASTN v2.10.1 (e-value: 1e-5) [40], and those with ≥60% identity and cumulative hit length ≥60% were filtered out. Finally, the contigs containing at least one telomere or SSU-rRNA gene, which had been filtered out, were reintroduced into the assemblies. Contigs with low coverage (contig coverage ranks <5%) were considered as micronuclear sequences and were deleted. The QUAST v5.2.0 [41] and BUSCO v5.2.2 [42] with the settings -l alveolata_odb10 --augustus --augustus_species tetrahymena were used to evaluate the final assemblies. Tandem Repeat Finder v4.10.0 and codetta v2.0 [43] were used to identify telomeres and infer the stop codon usage, respectively.

We used fastp v0.20.0 to trim the RNASeq data with the default parameters. The trimmed data was aligned to the reference genome of *E. coli* K12 MG1655 using Hisat2 v2.2.1 [44], and then removed the bacterial reads by Samtools v0.1.9. Trinity v2.21.0 [45] was used for reference-guided transcripts assembling. For *U. marinum* PJ20101A, the structural annotations were performed using EuGene v1.6.5 [46], with a Weight Array Matrix constructed from the strain’s own transcriptome. The stop codon of *U. marinum* PJ20101A was set to TGA only. For *C. vorax* PJ13002, both *de novo* gene prediction and transcriptome-based methods were applied. Reads were aligned to the genome using Hisat2 and converted to BAM format. These alignments were then used for gene structure prediction by Braker3 v3.0.8 [47] and StringTie v3.0.0 [48]. The transcripts assembled *de novo* and guided by the reference genome were combined and used as cDNA evidence to train the gene prediction model in Augustus v3.5.0 [49]. Additionally, EuGene was applied for annotation. Finally, all results from the structural annotation software were merged using in-lab Python scripts to get the final gene set. For functional annotation of the two ciliates, using BLASTP v2.9.0 (e-value 1e-5 -word_size 3 -num_alignments 20 -max_hsps 20 -show_gis), we annotated protein-coding genes (PCGs) with the non-redundant protein database (NR). GO and KEGG annotations were performed by OmicsBox v1.4.11 [50] and the KEGG pathway annotation was merged by the results of KAAS (KAAS–KEGG Automatic Annotation Server: https://www.genome.jp/kegg/kaas/; BBH method), and eggNOG-mapper v2 [51]. tRNAscan-SE v2.0.12 [52] was used to identify tRNAs, and rRNA genes in the genome were parsed using RNAmmer v1.2 with the settings -S euk [53]. Using the protein-coding sequences, codon usage analysis of the two strains was conducted with the seqinr v4.2.36 package in R.

Mitochondrial genome annotation was performed using GeSeq v2.03 (https://chlorobox.mpimp-golm.mpg.de/geseq.html), using genetic code 4 (The Mold, Protozoan, and Coelenterate Mitochondrial Code and the Mycoplasma/Spiroplasma Code) with added reference sequences from *Tetrahymena, Paramecium*, and *Uronema marinum* (NC_039174.1). PCGs underwent further verification using the NCBI Open Reading Frame Finder (https://www.ncbi.nlm.nih.gov/orffinder, accessed on 9 July 2025) and were functionally annotated via BLASTP searches against the NCBI non-redundant protein sequences (nr) database. rRNA genes were identified by BLASTN comparison with the rRNA genes of *Uronema marinum* (NCBI Gene IDs: 37625978, 37625943). tRNA genes were predicted using tRNAscan-SE v2.0 in default mode. Finally, telomere repeat regions were detected using Tandem Repeats Finder v4.10.0 [54].

### Comparative genomics and gene family analysis

Collinearity analysis within the genome of *U. marinum* PJ20101A was done first by BLASTP to find similar proteins with the E-value threshold of 1e-05. The function “File Merge For MCScanX” in TBtools was used to convert the gff3 to a concise format. Finally, genome collinearity results were generated using the Quick Run MCScanX Wrapper in TBtools-II v2.034. [55]. The collinearity visualization was done by the “Advanced Circos” function in TBtools [56]. Due to the fragmented genome of *C. vorax* PJ13002, with short contigs that contain very few genes, collinearity analysis was not feasible. Instead, we focused on analyzing its orthologous genes using OrthoFinder v2.5.4 [57].

To identify gene families, we obtained macronuclear genome sequences for 17 ciliate species: *Colpoda steinii* RZ4A (GWHERKZ00000000), *Glauconema* sp. LHA0827 (GWHDEDB00000000), *Halteria grandinella* QDHG01 (GCA_006369765.1), *Ichthyophthirius multifiliis* G5 (GCF_000220395.1), *Oxytricha trifallax* JRB310 (GCA_000295675.1), *Paramecium tetraurelia* d4-2 (GCA_000165425.1), *Pseudocohnilembus persalinus* 36N120E (GCA_001447515.1), *Stentor coeruleus* WM001 (GCA_001970955.1), *Stylonychia lemnae* 130c (GCA_000751175.1), *Tetrahymena thermophila* SB210 (GCA_000189635.1), *Euplotes vannus* EVANNUS_28419 (from the Ciliates Genome Database, http://ciliates.org/, accessed March 2018), and six additional species belonging to the Litostomatea class (*Lacrymaria* sp. QDB17S03; *Didinium* sp. QDB18S42; *Trachelophyllidae* sp. JNY21S103; *Monodinium* sp. JNY21S105; *Litonotus* sp. BJL23S26; *Dileptus* sp. BJL23S57; assembled by single-cell sequencing) [20]. In total, we got 453 685 protein sequences, comprising sequences derived from the 17 ciliate macronuclear genomes mentioned above and those encoded by the two ciliate genomes sequenced in this study. These protein sequences were used as the input of OrthoFinder v2.5.4 [57] to infer the phylogenetic orthology by the parameters: -f /input_folder -M msa -T fasttree -T 12 -a 12. We obtained three time points as primary calibrations from the Timetree database (http://www.timetree.org/; *Euplotes vannus* vs. *Paramecium tetraurelia*; *Tetrahymena thermophila* vs. *Paramecium tetraurelia*; *Ichthyophthirius multifiliis* vs. *Tetrahymena thermophila*). Then, r8s [58] was used to build the ultrametric tree based on the estimates provided by Timetree and the expansion and contraction analyses of gene families were performed using CAFE v5.1 [59]. Cafeplotter v0.2.0 (https://github.com/moshi4/CafePlotter, accessed on 22 September 2023) and iTOL v6 were then used to display and annotate the tree [60].

### RNAseq-based gene expression in different life stages

After sequencing, we obtained an average of 22.40 million and 18.52 million clean reads for each sample under predation and non-predation conditions of *C. vorax* PJ13002, respectively. The RNAseq clean reads from low-input RNA library constructions of different life stages were mapped to the genomes of *C. vorax* PJ13002 and *U. marinum* PJ20101A using Hisat2 v2.1.0. The resulting sam files were converted to bam format using Samtools. The expression level of each gene was calculated using StringTie v2.1.5 with the setting -e -B -G and the prepDE.py3 script [61]. Using DESeq2 v1.32.0 [62], genes with significantly different expression levels were identified with the setting |log_2_(FoldChange)| > = 1 and *P*_adj_ < .05. ClusterProfiler v4.0.2 was used to analyze the GO and KEGG pathway enrichments of the significantly differentially expressed genes, with *P*_adj_ < .05 and *q-value* < 0.05 [63].

### dN/dS estimation

Fastp v0.20.1 was used to trim adaptors and remove low-quality bases from raw reads of the two populations, then the above method was used to remove the food bacterial sequences. Clean reads were then aligned to the reference genomes using BWA v0.7.17 with default settings. Reads with multiple hits, which could be due to mis-mapping, were removed, and the SAM file was then converted to a BAM file by SAMtools ver. 0.1.9. Duplicates were marked and removed using Picard. Prior to selection analysis, alignment positions were stringently filtered using custom scripts to retain only sites with: (i) sequencing depth ≥ 2 for both forward and reverse reads; (ii) sample coverage >80% per site; (iii) cumulative gene coverage >80% of total length. Genes failing these thresholds were excluded. Estimates of nonsynonymous (dN) and synonymous (dS) substitution rates with standard errors were quantified for each gene using PAML v4.9’s yn00 program [64]. Codon alignments formatted for PHYLIP input were analyzed through configured yn00.ctl files (*C. vorax*: icode = 0; *U. marinum*: icode = 5). From the resulting dataset, ortholog pairs with dS = 0 or dS > 1 were discarded to avoid infinite dN/dS ratios. Final dN/dS values represent per-gene averages calculated across all qualifying sites.

## Results

### Predator–prey dynamics and control-threshold analysis reveal high biocontrol potential of *Uronema marinum* with *Chaenea vorax*

**Figure 1:**
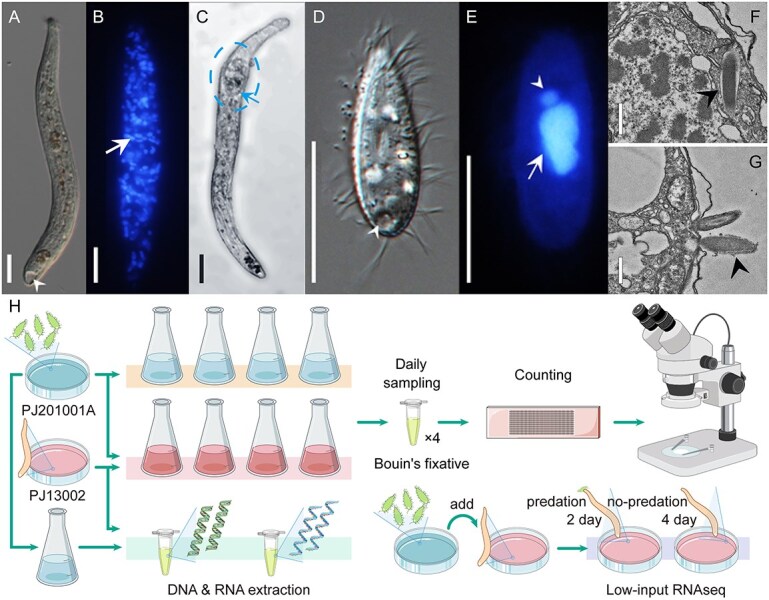
Morphology of the two ciliates and the workflow of this research. (A, D) *Chaenea vorax* PJ13002 and *Uronema marinum* PJ20101A *in vivo*. Arrowheads indicate contractile vacuoles (CV). (B, E) Macronucleus (arrows) and micronucleus (arrowhead) of *U. marinum* PJ20101A and *C. vorax* PJ13002 after Hoechst 33342 staining. (C) *Chaenea vorax* preying on *U. marinum*. The arrow shows an ingested *U. marinum* cell within the predator. (F, G) Intracellular or ejected trichocysts (arrowheads) in *U. marinum* observed by TEM. (H) The workflow of the predation experiment and subsequent molecular analyses, including nucleic acid extraction and low-input RNAseq. Scale bars: 20 μm (A–E); 0.5 μm (F, G).

*Chaenea vorax* (a raptorial predator) and *U. marinum* (a bacterivore) are both globally distributed ciliates in coastal waters that function as consumers and drive energy transfer within the microbial food web (Fig. 1) [65, 66]. *Chaenea vorax* has no record of parasitism, shows high morphological flexibility (Fig. 1A and B), and actively preys on *U. marinum* (Fig. 1C; Supplementary Video 1). By contrast, *U. marinum* is a dominant species, feeding on bacteria, dead tissue, and debris, and it can facultatively parasitize marine organisms, such as fish and shrimp (Fig. 1D) [24–26, 28]. Like other ciliates, it has both a macronucleus and a micronucleus (Fig. 1E).

**Figure 2:**
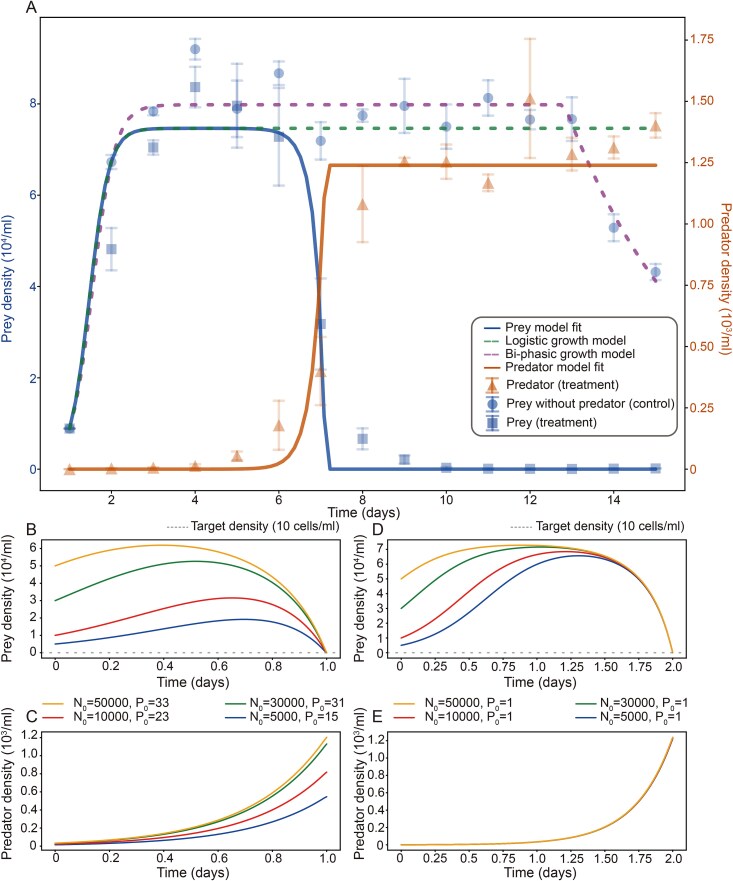
Predation dynamics of *C. vorax* (Predator) on *U. marinum* (Prey) and its potential for biocontrol. (A) Population dynamics from predation experiments at 18°C. Symbols represent experimental data (mean ± SE, *n* = 3) for the prey with predator (blue squares), prey without predator (blue circles) and the predator (orange triangles), and solid lines show the corresponding fits of the Rosenzweig–MacArthur model with a Holling type III functional response. The prey population in isolation (green/purple dashed line) is modeled by logistic/bi-phasic growth. (B, C) Simulated population trajectories required to achieve biocontrol, defined as reducing the prey population to be below 10 cells/ml within one day, under different initial predator (P_0_) and prey (N_0_) densities in cells/ml. (D, E) Simulated population trajectories required to achieve biocontrol effects within 2 days.

To estimate the efficiency of predation, we first performed feeding experiments (Fig. 1H), using *C. vorax* PJ13002 and *U. marinum* PJ20101A. At 18°C—comparable to aquaculture pond temperatures where *Uronema* outbreaks often occur—*C. vorax* PJ13002 significantly reduced *U. marinum* PJ20101A populations, with the strongest effect observed on Days 6–8 (Fig. 2A; Supplementary Table S2). These provided the basis for subsequent mechanistic modeling. To mechanistically quantify the associated population dynamics, we fit prey without predator trajectories first. A logistic model with a decay term was fitted to the experimental data of prey growth alone, yielding estimates for intrinsic growth rate ($\hat{r}\approx 4.24$ day^−1^ in *U. marinum* vs. 2.20 day^−1^ in *C. vorax*) and carrying capacity ($\hat{K}\approx 7.46\times{10}^4$ cells/ml), with fit quality ${R}^2=0.66$, capturing early growth but underestimating late-stage decline. A bi-phasic extension introducing a density-independent decay term (${\hat{t}}_s\approx 12.8$ day and $\hat{k}\approx 0.30$ day^−1^), improves fit substantially (${R}^2=0.95$). We therefore retain the bi-phasic form only as a descriptive account of prey without predator, while fixing the logistic estimates ($\hat{r},\hat{K}$) for subsequent predator–prey analysis to avoid confounding environment-driven mortality with predation effects (Fig. 2).

We further quantified the predation dynamics using predator–prey modeling based on the Rosenzweig–MacArthur framework with a Holling type III functional response. The sigmoidal form of the Holling type III functional response is consistent with density-dependent predation, wherein predation efficiency appears to increase at higher prey densities, but decreases at lower densities, possibly reflecting prey refuge effects (e.g. spatial refuge in the water column for *U. marinum*). This mechanistic interpretation aligns with established ecological theory regarding predator–prey dynamics [30], particularly the conditions that promote Type III functional responses via effective prey refuges, which have been documented in protistan systems [67]. As in classical Rosenzweig–MacArthur formulations, the model assumes a single prey species and no alternative food resources are available to the predator. The two-step strategy (prey without predator first, then full dynamics) increases parameter identifiability by separating intrinsic prey growth from predation terms. The exceptionally high explanatory power of the model (*R*^2^ = 0.97 for prey, *R*^2^ = 0.94 for predator) suggests that it effectively captures key aspects of the biological interaction. Details of model formulation, numerical integration, and parameter estimation are provided in Materials and Methods.

To be specific, the estimated parameters describe *C. vorax* as a predator with a suite of distinctive traits. Its attack rate ($\hat{a}$= 0.073 ml predator^−1^ day^−1^), which reflects per-capita encounter and capture efficiency, is comparable to or exceeds that of many other efficient microbial predators, while falling within the range expected for a protist of its size based on allometric relationships [68, 69]. This indicates effective prey search and capture, as supported by the phase of rapid prey suppression observed at 6–8 days in our predation experiments (Fig. 2A). The handling time ($\hat{h}$= 0.005 day ≈ 7.2 min) is shorter than that of some protozoan predators [12] and is also consistent with size-based predictions for raptorial feeders [69]. This combination of traits indicates efficient prey processing, potentially aided by proteolysis—a notion consistent with microscopic observations of rapid prey dissolution inside the predator. The conversion efficiency ($\hat{e}$= 0.011) reflects a trophic strategy emphasizing predation activity over biomass accumulation, consistent with observations that predatory ciliates allocate less energy to growth than algivorous taxa [70]. The estimated low death rate ($\hat{\beta}$= 0.002 day^−1^) is orders of magnitude lower than the median for planktonic ciliates (~0.62 day^−1^), which might result from its high degree of starvation resilience [69, 71]. This model-derived inference is consistent with our experimental observation that *C. vorax* can survive for extended periods under low-prey conditions in the culture system (Fig. 2A). The combination of an efficient attack, rapid handling, moderate metabolic demand, and high endurance results in a dynamic that may position *C. vorax* as a promising candidate for biocontrol, suggesting an ability to maintain persistent suppression pressure without rapid self-amplification.

To explore the applied potential of *C. vorax* as a biocontrol agent against *U. marinum*, we performed control threshold analysis based on the fitted model. We define the minimal predator inoculum, ${P}_0^{min}$, as the lowest initial predator density capable of suppressing prey populations below a threshold ${N}^{\ast }$ within a finite time horizon $T$. This was computed via binary search combined with ODE integration of the fitted model, comparing terminal prey densities $N(T)$ against suppression thresholds. Simulations suggested that even under high initial prey densities (5000–50 000 cells/ml), the introduction of relatively low densities of *C. vorax* (as low as 1–33 cells/ml) could lead to a reduction exceeding 99.8% within 1–2 days (Fig. 2B–E). The set of simulated scenarios and predicted inoculum thresholds is listed in Supplementary Table S3, and simulation procedures are detailed in Materials and Methods. These results indicate that *C. vorax* exerts powerful control potential, capable of limiting *U. marinum* outbreaks with relatively low inoculum size. Moreover, the framework provides a flexible predictive tool. By varying prey density, suppression efficiency, and time horizon, we could estimate operational inoculation requirements under diverse scenarios. Collectively, these results suggest that the predator–prey modeling framework provides a useful basis for interpreting the mechanistic features of *C. vorax* predation and for exploring its potential application in biological control of facultative ciliate pathogens in aquaculture systems.

### Predation-associated pathways are numerous in the macronuclear genome of *C. vorax*

**Figure 3:**
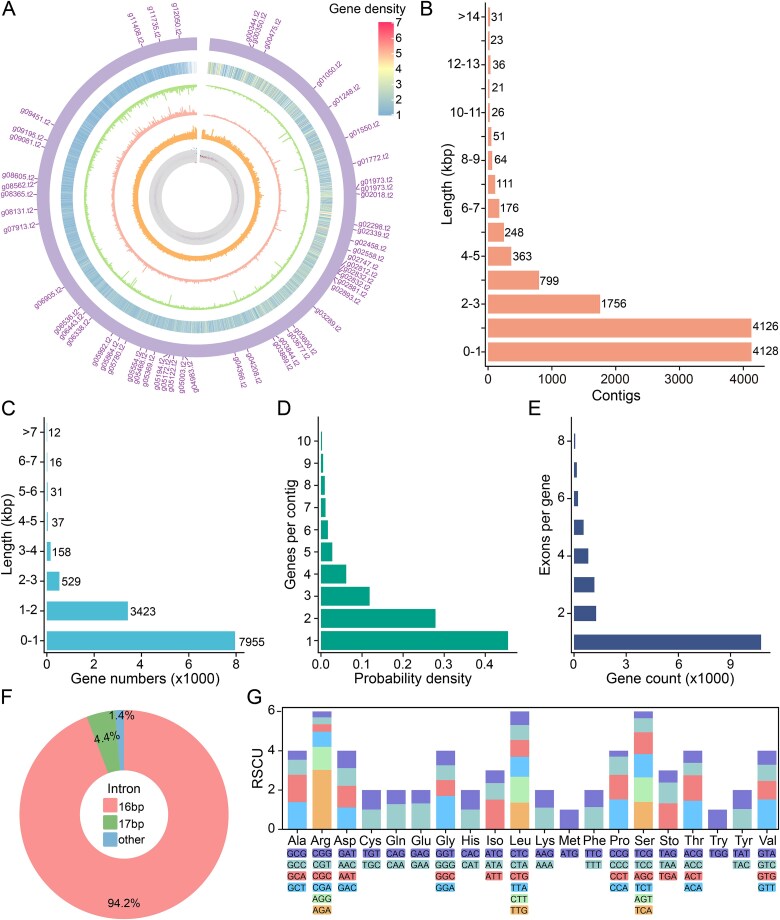
Features of the *C. vorax* PJ13002 *de novo* macronuclear assembly. (A) Circular visualization of genomic features (outermost to innermost): concatenated contigs with annotated genes showing alternative translation initiation sites at shared loci; gene density; Nanopore sequencing depth; Illumina sequencing depth; GC content; GC skew. The GC content and GC skew were calculated in 1000 bp sliding windows (500 bp step size). (B) Distribution of contig length. (C) Distribution of gene length with introns. (D) Distribution of gene number per contig. (E) Distribution of exons per gene. (F) Distribution of intron length. (G) Relative synonymous codon usage (RSCU) distribution. Sto represents stop codons.

Based on Nanopore long-read sequencing, we *de novo* assembled the macronuclear genome of *C. vorax* PJ13002, with 23.49 Mbp in size, 11 959 contigs, no gaps, GC content of ~40%, and a N50 of 2765 bp (Table 1; Fig. 3A). Most contigs were ≤ 2000 bp in length, accounting for 69% of the total (Fig. 3B). Among the contigs, 5153 contained telomeric repeats ([C_4_A_2_T]_n_) at both ends, and 4533 contigs contained only one telomere, together accounting for 81% of the total, indicating a highly-fragmented genome with gene-sized chromosomes, typical of haptorid ciliates. 2867 of the nanochromosomes carry only one gene. 12 212 PCGs were annotated, with a mean length of 1051 bp (Fig. 3C, D).

**Table 1:** Features of the two *de novo* macronuclear assemblies.

Species	*Uronema marinum* PJ20101	*Chaenea vorax* PJ13002
Genome size (bp)	95 371 444	23 490 212
Contig number	143	11 959
GC (%)	17.30	40.04
N50	1 099 901	2765
L50	26	2246
Protein-coding genes number	28 748	13 084
Mean gene length (bp)	2951	1192
Mean exon length (bp)	498	574
Median intron length (bp)	75	16
tRNA number	346	30
rRNA number	3	3
BUSCO score (protein)	92.4%	69.0%

Many of these genes are involved in prey capture, phagocytosis, and digestion (Supplementary Table S4). For instance, structural components such as actin and regulatory elements including calmodulin, calcium-dependent protein kinases, and inositol 1,4,5-triphosphate receptors emphasize the central role of calcium-triggered cytoskeletal remodeling in prey capture. Likewise, proteolytic enzymes (e.g. leishmanolysin) and processing enzymes such as proprotein convertase are essential for degrading captured prey within phagolysosomes. The co-occurrence of rapid calcium signaling effectors, actin-dynamics regulators, and proteolytic enzymes together provides a molecular basis that could support predatory ability of *C. vorax*, which is consistent with its role as a microbial predator in its environment.

10 755 genes (88%) lacked introns, which were among the shortest in ciliates, predominantly 16 bp in length (94% of all introns), with the remaining introns being mostly 17 bp (Fig. 3E, F). Codon usage in the *C. vorax* genome shows a balanced AT–GC distribution, with GAA (Glu) and AAA (Lys) as the most frequent codons, and no extreme preferences (maximum relative synonymous codon usage (RSCU) = 3.09 for AGA; Fig. 3G). *C. vorax* frequently uses GC-rich codons, shows more even synonymous codon usage (GC content 40.0%), and retains all three standard stop codons without reassignment, different from most other ciliates. The mitochondrial assembly of *C. vorax* PJ13002 (19 249 bp) contains 22 PCGs and 3 tRNA genes (Supplementary Fig. S1; Supplementary Table S5).

### Ciliary movement and digestion genes in *C. vorax* are differentially expressed upon predation

**Figure 4:**
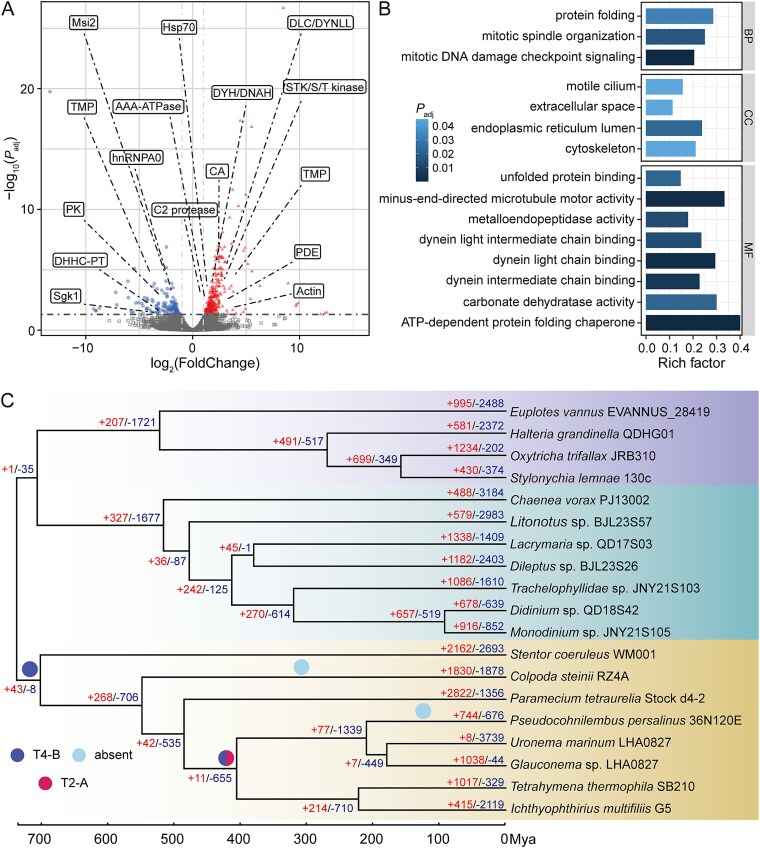
Transcriptomic and comparative genomic analyses of *C. vorax* PJ13002. (A) Differential gene expression between predatory and non-predatory states, with red triangles representing significantly up-regulated genes and blue circles indicating down-regulated genes (log₂(FoldChange) >1 or < −1; *P*_adj_ < .05). (B) Gene Ontology analysis of significantly up-regulated genes in predatory states, categorized by Biological Process (BP), Cellular Component (CC), and Molecular Function (MF). (C) The dynamic evolution of gene families in 19 ciliates was analyzed (similarity between genes was determined through all-to-all comparisons). The bottom numbers represent the estimated time in millions of years (based on the relevant rate clock). The positive (red) and negative (blue) numbers above each branch indicate the number of gene families that expanded or contracted in the species. Blue and red solid circles denote T2-A (hap_ctg.000003Fg0024851) and T4-B (hap_ctg.000025Fg0106551) trichocyst-matrix proteins.

To investigate the molecular mechanisms underlying the predatory behavior of *C. vorax*, we performed low-input RNAseq on cells in predation vs. non-predation, and differential gene expression analysis, identifying 350 significantly up-regulated genes and 135 down-regulated genes in predation compared to non-predation (Fig. 4A; Supplementary Table S6). The gene enrichment analysis (*P*_adj_ < .05) for the significantly down-regulated genes revealed that only two biological processes, namely regulation of gene expression and reciprocal meiotic recombination, were significantly enriched (Supplementary Table S7). Consistent with this, KEGG analysis showed downregulation of genes related to RNA binding and post-transcriptional regulation, calcium signaling, and protein modification (Supplementary Table S7). These findings might suggest a strategic redistribution of energy resources away from non-essential processes—such as genetic information processing and sustained signaling—toward immediate predation-related functions, reflecting a suppression of basal cellular activities during active feeding.

350 up-regulated genes were enriched in diverse molecular functions including ATP-dependent protein folding chaperone activity, minus-end-directed microtubule motor activity, dynein chain binding, metalloendopeptidase activity, and carbonate dehydratase activity. These genes were also enriched in cellular components such as extracellular space, cytoskeleton, and motile cilium, and in the protein folding process (Fig. 4B; Supplementary Table S7). This indicates that the concurrent expression of genes related to cellular proteolysis, microtubule-based phagosomal transport, and stress-resilient proteostasis are consistent with a coordinated process for prey processing in *C. vorax*.

Although specific biological processes—including calcium ion transmembrane transport, carbohydrate metabolic processes, protein catabolic processes, transmembrane transport, lipid metabolism, lyase activity, endonuclease activity, endopeptidase activity, and hydrolase activity—did not reach statistical significance in this stringent enrichment analysis, their frequent representation among the differentially expressed genes (particularly the upregulated genes) still suggests their close association with predation behavior (Supplementary Table S7). These processes may be involved in digestion of the prey (*U. marinum*), such as phagosome maturation, digestion of prey-derived macromolecules (e.g. proteins, nucleic acids), and nutrient absorption/utilization. Complementing the GO enrichment findings, the results of the KEGG enrichment analysis (*P*_value_ < .05) also confirmed the above main processes related to dynein proteins (K10408: dynein axonemal heavy chain) and digestion (K01404: leishmanolysin).

Pathways of proprotein convertase subtilisin/kexin type 5 and RNA-binding protein Musashi appeared in both up- and down-regulated gene sets, indicating their possible pleiotropy in regulating different pathways during predation. Although transcriptionally suppressed, both genes are associated with broader predation-relevant pathways: protein processing and digestive enzyme activation, and post-transcriptional regulation of rapid-response genes. Their dual enrichment suggests a shift from general cellular activities toward targeted functions essential for predation, highlighting the metabolic reallocation underlying predatory behavior in *C. vorax*.

### Trichocysts may contribute to the defense of *Uronema marinum* against *Chaenea vorax*

As a dominant ciliate inhabiting coastal environments, *U. marinum* faces persistent predation pressure, which may have driven the evolution of various defense mechanisms. To find out the molecular basis of these mechanisms, the macronuclear genome of *U. marinum* PJ20101A was first *de novo* assembled, with 95.37 Mbp in size (Table 1). This AT-rich genome (GC ~17%) encodes 28 748 PCGs (details are in Supplementary Text 1, Supplementary Figs. S1, S2 and Supplementary Tables S8–S10).

**Figure 5:**
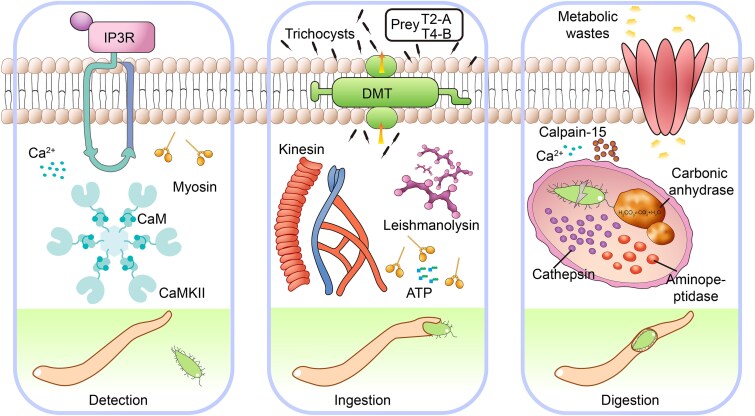
Molecular basis of predation in the marine ciliate *C. vorax* PJ13002. The schematic illustrates key molecular events proposed to mediate predation, beginning with prey detection where calcium signaling (Ca^2+^) is suggested to trigger a contractile machinery involving myosin, CaM, and CaMKII, thereby initiating the predation process. Prey ingestion appears to be driven by motor proteins, which may also utilize the DMT efflux pump to expel trichocysts toward the prey, followed by a digestion process potentially mediated by effectors such as leishmanolysin and calpain-15 for proteolysis, along with digestive enzymes including carbonic anhydrase, cathepsin, and aminopeptidase, prior to the final release of metabolic wastes.

To investigate whether certain genomic features are associated with defensive responses during predation, we performed transcriptomic analysis on *U. marinum* cells being swallowed by *C. vorax*. After accounting for potential cross-species homology by filtering shared gene families (Supplementary Fig. S3), we identified 28 genes expressed in *U. marinum* (*P*_value_ < .05; Supplementary Table S11). These include genes encoding ribosomal proteins, HSP70, trichocyst matrix proteins (T2-A and T4-B; Fig. 5), histone-fold proteins, among others. Trichocysts are membrane-bound cortical organelles that, upon stimulation, undergo exocytosis to eject rigid, proteinaceous shafts. This explosive discharge is a well-characterized mechanism for deterrence against predators [72, 73]. Therefore, when *U. marinum* encounters predation pressure from *C. vorax*, it may activate the trichocyst secretion pathway, potentially representing an attempt to release trichocyst matrix proteins as part of an active defense mechanism to resist attack or interfere with the ingestion process. Our TEM observations provide support for this, as trichocysts were clearly present in the cortex of *U. marinum* (Fig. 1F and G). Nevertheless, the high predation efficiency of *C. vorax* suggests that it can overcome this and other potential defensive strategies. *Uronema marinum* persists as a dominant coastal species largely due to its r-selected life history strategy, characterized by one of the fastest intrinsic growth rates among ciliates, with a doubling time of ~3.9 h ($\hat{r}\approx 4.24$ day^−1^, measured at a suboptimal temperature of 18°C), rivaling that of the fast-growing model ciliate *Tetrahymena thermophila* (2–5 h) and markedly exceeding that of *Paramecium tetraurelia* (8–9 h) [74–79].

### Molecular evolution of predation-associated genes

Gene family analysis of *C. vorax* PJ13002, the prey *U. marinum* PJ20101A, and 17 other ciliates (See details in Materials and Methods) identified 48 186 gene families. The analysis of gene family expansion and contraction revealed eight expanded and 3739 contracted families of *U. marinum*, while *C. vorax* showed 488 expanded and 3184 contracted families (Fig. 4C). The analysis revealed an ancient origin for the *C. vorax* lineage, which diverged early from other haptorian ciliates. This phylogenetic placement, consistent with prior work [21, 80, 81], further showed that the *U. marinum* lineage diverged much later and is closely related to *Glauconema trihymene*. The extensive gene family contraction in *C. vorax* (3184 families) likely reflects its exceptionally early divergence time and the degree of genetic divergence from other ciliates (Fig. 4C).

The separate expanded gene families in *C. vorax* demonstrate relevance with predation, integrating sensory perception, attack mechanisms, and metabolic adaptations (Supplementary Fig. S4A; Supplementary Table S12). Enhanced mechanosensory perception and chemosensory behavior support prey localization, and actin-driven contractility and ciliary motility contribute to target engagement [82]. The cellular key protein hydrolysis mediated by lysosomal enzymes and calcium-gated membrane channels ensures the effective absorption by the predator, and then the absorption of nutrients is achieved through specialized amino acid and carboxylic acid transport proteins. The efficiency of these integrated systems may be enhanced by adaptations to the benthic environment (*C. vorax* is a periphytic ciliate). For instance, proton-transporting membrane networks could aid in pH and ion homeostasis within the variable sediment-water interface, and oxygen-responsive pathways may support metabolic flexibility in oxygen-poor benthic environment. Concurrently, calcium-regulated signaling coordinates rapid predatory strikes through cytoskeletal contractility, optimizing attack precision. The evolution of *C. vorax* may involve ciliary-based prey capture mechanisms, potential heterochromatin-associated regulation of prey-response genes, and specialized membrane microdomain reorganization—suggestive of molecular toolkits that collectively could establish *C. vorax* as a highly efficient microbivore, potentially through integrated innovations in sensor-effector coordination, digestive specialization, and toxin resistance. These results are consistent with the expansion of the gene families to which these predation-associated, up-regulated genes belong (Supplementary Tables S6, S7, and  S12; Fig. 4C; Supplementary Fig. S4A).

The KEGG enrichment analysis also confirmed that the expanded gene families of *C. vorax* are functionally beneficial for predatory behavior (Supplementary Fig. S4B; Supplementary Table S12). The calcium-signaling components (calmodulin; CaMKII; IP3 receptor) drive contraction-based attacks, and extracellular proteases (leishmanolysin; calpain-15) degrade prey tissues. Specialized molecular pumps (MATE transporter; ABCA3) simultaneously expel ingested toxins, and the energy sensor AMPKα optimizes post-capture metabolism. Rab9-mediated trafficking further refines digestive organelle maturation, coupled with Musashi protein enabling rapid genetic responses to prey cues. These co-expressions are consistent with a potential multi-step predatory process involving attack, degradation, and detoxification pathways.

Given the extremely long evolutionary history of ciliates, evolutionary inferences based on gene family expansion/contraction analyses may be inaccurate and lack resolution. We thus conducted population genomic analyses on natural strains of both *C. vorax* (20 strains) and *U. marinum* (seven strains) newly collected in this study (Supplementary Table S13). Population genetic analyses revealed a substantially higher nucleotide diversity in the predator, with *C. vorax* demonstrating a π of 0.063 and a four-fold degenerate site π (π_s_) of 0.187, a level markedly exceeding that reported for most other ciliates [83–85]. After excluding genes with poor alignment quality, dS = 0, or dS > 1, we obtained analyzable dN/dS ratios for 11 differentially expressed genes (DEGs) during predation in *C. vorax* (Supplementary Table S6). These genes all had dN/dS ratios significantly less than 1 (median = 0.072, mean = 0.080, SE = 0.059), consistent with generally relaxed selection on predator-associated genes.

We also did a population genomic analysis of *U. marinum* during predation, which possesses much lower genetic diversity (π = 0.009; π_s_ = 0.045) than the predator, and identified no genes with a dN/dS ratio > 1 among the nine DEGs analyzed (Supplementary Table S11). Four genes—ribosomal protein L23/L15e core domain, ribosomal protein S5 domain two-type fold, hypothetical protein PPERSA_10365, and trichocyst matrix protein T4-B—showed dN = 0 (dN/dS = 0), indicating extreme functional constraints. Another trichocyst-related gene, T2-A, also showed a low dN/dS value of 0.039 (Supplementary Table S11). The lack of positive selection and evidence of strong purifying selection on multiple defense genes (including four under extreme constraint and T2-A with dN/dS = 0.039) suggest pronounced purifying selection on defense-related loci in the prey, possibly reflecting functional conservation or limited standing variation among natural strains.

## Discussion

This study establishes the marine ciliate *C. vorax* as a highly efficient predator and provides a comprehensive molecular dissection of its predatory traits through an integrated ecological, multi-omic, and evolutionary approach. This high predatory efficiency aligns with and provides a physiological basis for early laboratory observations that established *C. vorax* as a predator capable of decimating populations of *Cyclidium* sp. [86]. We quantified and modeled its predation dynamics against *U. marinum*, assembled and annotated its macronuclear genome, identified key predation-related pathways and gene families, and revealed dynamic transcriptomic reprogramming during predation. Furthermore, population genomic analyses revealed contrasting evolutionary constraints between predator and prey. *C. vorax* showed signatures of relaxed selection on predation-related genes, indicative of trophic flexibility—a trait consistent with its previously reported ability to capture and utilize diverse prey, particularly those with a long caudal cilium [86]. This flexibility is further supported by our observations of *C. vorax* preying on *Miamiensis avidus* and *Metanophrys* sp. (also common aquaculture pathogens; Supplementary Fig. S5; Supplementary Videos 2, 3).

These divergent selective patterns—marked by much higher genetic diversity in the predator (π = 0.063) than in the prey (π = 0.009)—suggest an imbalance that could drive an asymmetric evolutionary arms race. The predator, with its greater standing genetic variation, may adapt and innovate more rapidly in response to prey defenses, whereas the prey lags behind. Nevertheless, the prey may still persist in the long term by relying on its r-selected life history strategy, prioritizing rapid reproduction over evolutionary novelty. This propensity for fast reproduction could also contribute to the success of many opportunistic pathogens and frequent dominant species in aquaculture systems.

The Rosenzweig–MacArthur predator–prey model has the potential to guide the application of *C. vorax* as a biocontrol strain against the facultative pathogen *U. marinum*, particularly by restoring natural trophic interactions in aquaculture systems and thereby promoting sustainable, green aquaculture. This model links several key operational variables—such as initial prey density, desired suppression efficiency, and required time to control—to estimate the suitable predator inoculation densities. These findings suggest the possibility of using *C. vorax* as a biocontrol strain against *U. marinum*—a notoriously resilient aquaculture pathogen—and provide a theoretical basis for its practical application using density-based intervention strategies. Moreover, the modeling framework introduced here may be transferable; with appropriate experimental data on predator–prey interactions, it could potentially be adapted to estimate inoculation densities for controlling other microbial pathogens in aquaculture, providing a flexible tool for designing targeted and sustainable biocontrol strategies. Future extensions incorporating ecologically relevant predator traits—such as body-size plasticity and prey selectivity—could further improve predictive realism and enhance its utility for optimizing biocontrol performance under diverse environmental conditions [87].

Despite the insights gained from this study, there are several limitations. Functional validation of key predation-associated genes—those involved in cytoskeletal remodeling, proteolysis, and toxin resistance—is still required to confirm their roles; however, efficient gene-editing tools for *C. vorax* are not yet available. Nanosphere-based technology may help address this gap by delivering RNA strands into cells to enable transient gene manipulation such as knockdown or overexpression, and may even facilitate direct DNA editing for permanent genetic modifications [88–90]. Moreover, the mechanism behind the failure of *U. marinum*’s trichocyst-based defenses against *C. vorax* warrants further cytological and molecular investigation. Another concern is the relatively low assembly completeness of the *C. vorax* genome, potentially resulting from technical constraints (e.g. suboptimal DNA quality, library construction issues, sequencing biases/variability, and other factors), inherent genomic complexity, and the documented difficulties of standard assemblers in handling polyploidy. These issues may collectively compromise the accuracy and comprehensiveness of subsequent genomic analyses. Furthermore, the limited population strains for both predator and prey reduce the statistical power to infer population parameters and detect signatures of selection. Additionally, although we observed predation activity against other aquaculture-relevant pathogens, namely *Miamiensis avidus* and *Metanophrys* sp., systematic explorations of *C. vorax* against various facultative ciliate pathogens are needed. A comprehensive assessment of its practical potential could include a systematic comparison of the predation efficiency of *C. vorax* against other predatory ciliates known to target *U. marinum*, such as *Acineria incurvata* (Supplementary Fig. S6). In parallel, the modeling framework introduced here can be further refined by incorporating data from multiple predator and prey species, thereby enabling comparative evaluations of different biocontrol strategies and identifying optimal predator–prey assemblages to support effective aquaculture management. Finally, the translation of *C. vorax* into a reliable biocontrol agent faces practical challenges, including its large-scale cultivation, physiological stability across varying environmental conditions (e.g. temperature, salinity, pH, bacterial composition, culturing space, and other factors), and the need to fully evaluate its prey range and differential predation efficiency against various aquaculture pathogens. A comprehensive assessment must also consider the temporal dynamics of its predation activity, potential variability in performance across different aquaculture systems, and the influence of spatial heterogeneity on predator–prey interactions.

The high predation efficiency of *C. vorax*, as quantified in our predation experiments, may be attributed to a synergistic system that couples a pre-adapted genomic repertoire with dynamic transcriptional reprogramming. This integrated framework coordinates rapid prey capture, robust digestion, efficient resource allocation, among other processes. It is plausible that the marine environment, characterized by high ionic strength and viscosity, has shaped specific molecular adaptations in this system [91]. *Chaenea vorax* uses a calcium-myosin contractility system to capture prey. During this process, calcium signals (such as IP3 receptors and CaMKII) activate cytoskeletal remodeling, thereby immobilizing the prey. These related genes are similar to those found in previous studies on other predatory ciliates [21, 92]. Moreover, this mechanism is functionally convergent with the contractile rings of nematode-trapping fungi like *Drechslerella stenobrocha*, although their evolutionary origins are different [93]. Following capture, lysosomal proteases (e.g. leishmanolysin, calpain-15) are used for cellular hydrolysis of prey components. The efficacy of these hydrolytic enzymes may be particularly adapted to the ionic composition and pH typical of marine phagolysosomes. Our transcriptomic analysis revealed significant upregulation of a drug/metabolite transporter (DMT) in *C. vorax*. We propose that it functions as an efflux pump to expel ingested toxins—a mechanism that, although well-established in bacteria, is also widely present among eukaryotes [94–96]. This finding suggests that *C. vorax* may use active detoxification to counteract defensive structures such as trichocysts released by *U. marinum*, highlighting a sophisticated counteradaptation within this microbial predator–prey arms race.

The concomitant up-regulation of carbonate dehydratase—a key pH regulator—implies coordination of lysosomal acidification during digestion. This pH regulation could be critical for maintaining enzymatic activity under the buffering conditions of seawater. The critical role of lysosomal activation is found in diverse predatory systems, from the rapid permeabilization of cyanobacterial prey by *Pseudomicrothorax dubius* [97] to amoebic trogocytosis in *Entamoeba histolytica* [98]. From an applied perspective, the potent cellular proteases of *C. vorax* could inspire solutions for disrupting resistant cells; developing cell lysis buffers incorporating these enzymes might facilitate DNA extraction from recalcitrant microbial prey, offering a valuable tool for future laboratory protocols and biotechnological applications. Therefore, although the core molecular machinery for predation might be conserved across aquatic environments, its regulation and expression in *C. vorax* appear to be adaptively tailored to the unique challenges of the marine habitat.

Under predation pressure from *C. vorax*, the prey *U. marinum* shows significant expression of trichocyst matrix protein, suggesting a defensive response through the release of trichocysts. This finding is consistent with the conserved defensive function of trichocysts in many ciliates: the matrix proteins in them can aggregate into filaments upon stimulation, paralyzing the enemy or hindering the feeding process [72, 99–102]. Gene family analyses further reveal that trichocyst-related genes are broadly conserved across bacterivorous Oligohymenophorea (e.g. *Tetrahymena, Paramecium*), yet conspicuously absent in predatory ciliates like *C. vorax* and other members in Litostomatea (Fig. 4C). The trichocyst matrix protein T2-A appears only in four species including *U. marinum*, and trichocyst matrix protein T4-B occurs widely across Oligohymenophorea (except the soil-adapted *C. steinii* and the marine ciliate *Pseudocohnilembus persalinus*), highlighting evolutionary divergence in defense mechanisms tailored to specific ecological pressures (Fig. 4C).

This study establishes a marine ciliate predator–prey system and shows the molecular basis of the predation process (Fig. 5). Genomic evidence reveals that *C. vorax* has diverse predation effectors, supporting calcium-triggered contractility, cellular proteolysis, and toxin expulsion. Evolutionary analyses further reveal both deep divergence and high genetic diversity, consistent with long-term adaptation to predatory lifestyles. Although prey such as *U. marinum* uses defenses that are largely ineffective, their r-selected life history strategy ensures persistence in predator-rich environments. Taken together, these findings advance our understanding of the molecular and evolutionary basis of ciliate predation and open promising avenues for developing sustainable biocontrol strategies in aquaculture. The establishment of this system provides a foundation to pursue further questions, including the ecological impacts of ciliate predation on microbial networks, the molecular mechanisms of prey recognition, and the potential for engineering predatory phenotypes for targeted applications.

## Supplementary Material

wrag053_Supplemental_Files

## Data access

All data generated or analyzed during this study are included in this article and its Supplementary material files. Sequence data of *C. vorax* PJ13002/C13, *U. marinum* PJ20101A, and the natural strains for population-level analyses are available at the BioProject (PRJNA1166914) of NCBI. Two newly assembled and annotated macronuclear genomes are uploaded to the NGDC: GWHGRYR00000000.1 (*Uronema marinum* PJ20101A) and GWHGRYS00000000.1 (*Cheanea vorax* PJ13002). The GenBank accession numbers of the 18S rRNA gene sequences of the strains were OP050455.1 (*U. marinum* PJ20101A), ON989824.1 (*C. vorax* PJ13002), and PQ358406.1 (*C. vorax* C13). All scripts and input data for plotting and statistical analyses are in our lab’s GitHub (https://github.com/IEMB-LEG/Biocontrol-potential-and-molecular-basis-of-predation-in-a-marine-raptorial-ciliate/tree/main; the Zenodo DOI is https://doi.org/10.5281/zenodo.18149410).
