# Supplementary material for: Biocontrol potential and molecular basis of predation in a marine raptorial ciliate
Source: ISME J. 2026 Mar 13;20(1):wrag053. doi: 10.1093/ismejo/wrag053 (PMC13096750; doi:10.1093/ismejo/wrag053)
Supplement: wrag053_Supplemental_Files [file wrag053_supplemental_files.zip › Supplementary_Figures_for_ISME_-_20260310_wrag053.docx]

**Biocontrol potential and molecular basis of predation in a marine raptorial ciliate**

Jiao Pan^1,2^, Jiahao Ni^1^, Yaohai Wang^1^, Ziguang Deng^1^, Hongwei Yue^1^, Kangqiao Dong^3^, Yichen Li^1^, Zhongze Lei^1^, Ziming Ma^1^, Gongze Hu^1^, Runda Chi^1^, Zhongyu Chang^1^, Qikai Chen^1^, Yujun Cai^1^, Hanlin Shen^1^, Runzhi Shi^1^, Wei Yang^1^, Xinpeng Fan^3^, Weiyi Li^4^, Zhiqiang Ye^5^, Michael Lynch^6^, Yu Zhang^7,*^, Hongan Long^1,2,*^

^1^ Key Laboratory of Evolution and Marine Biodiversity (Ministry of Education), Institute of Evolution and Marine Biodiversity, Ocean University of China, Qingdao, Shandong Province, China 266003

^2^ Laboratory for Marine Biology and Biotechnology, Qingdao Marine Science and Technology Center, Qingdao, Shandong Province, China 266237

^3^ School of Life Sciences, East China Normal University, Shanghai, China 200241

^4^ Department of Genetics, Stanford University School of Medicine, Stanford CA, USA 94305

^5^ School of Life Sciences, Central China Normal University, Wuhan, Hubei Province, China 430079

^6^ Biodesign Center for Mechanisms of Evolution, Arizona State University, Tempe AZ, USA 85287

^7^ School of Mathematics Science, Ocean University of China, Qingdao, Shandong Province, China 266000

**Corresponding authors**

* To whom correspondence may be addressed.

Hongan Long

[longhongan@ouc.edu.cn](mailto:longhongan@ouc.edu.cn)

Key Laboratory of Evolution and Marine Biodiversity (Ministry of Education), Institute of Evolution and Marine Biodiversity, Ocean University of China

Laboratory for Marine Biology and Biotechnology, Qingdao Marine Science and Technology Center

No. 5 Yushan Road

Qingdao, Shandong Province 266003

China

Yu Zhang

[zhangyu6929@ouc.edu.cn](mailto:zhangyu6929@ouc.edu.cn)

School of Mathematics Science, Ocean University of China

No. 238 Songling Road

Qingdao, Shandong Province 266000

China

Short title: Ciliate biocontrol potential & predation

**Supplementary Figures**


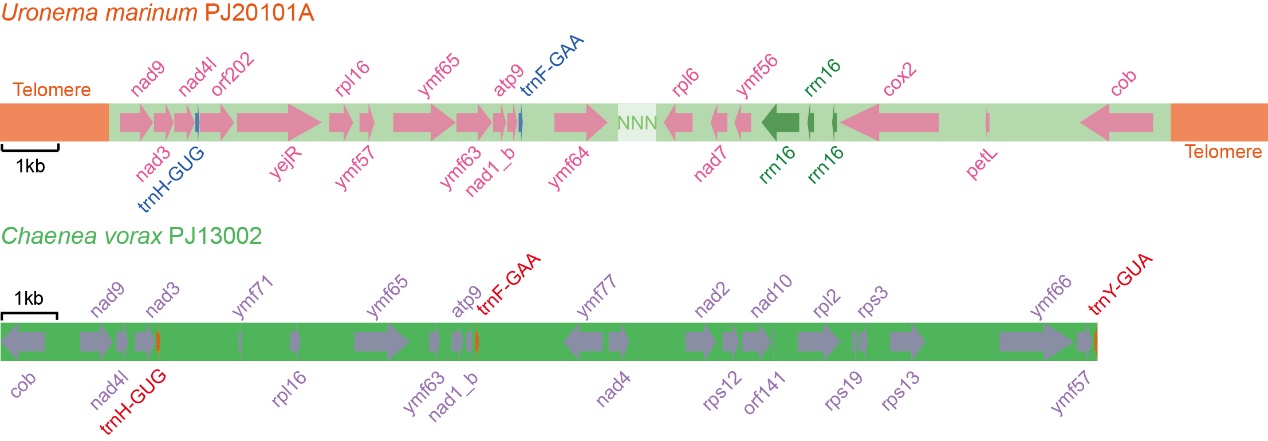


**Supplementary Fig. S1. The assembly, annotation, and telomere positions of the mitochondrial genomes of *U. marinum* PJ20101A and *C. vorax* PJ13002.**

**
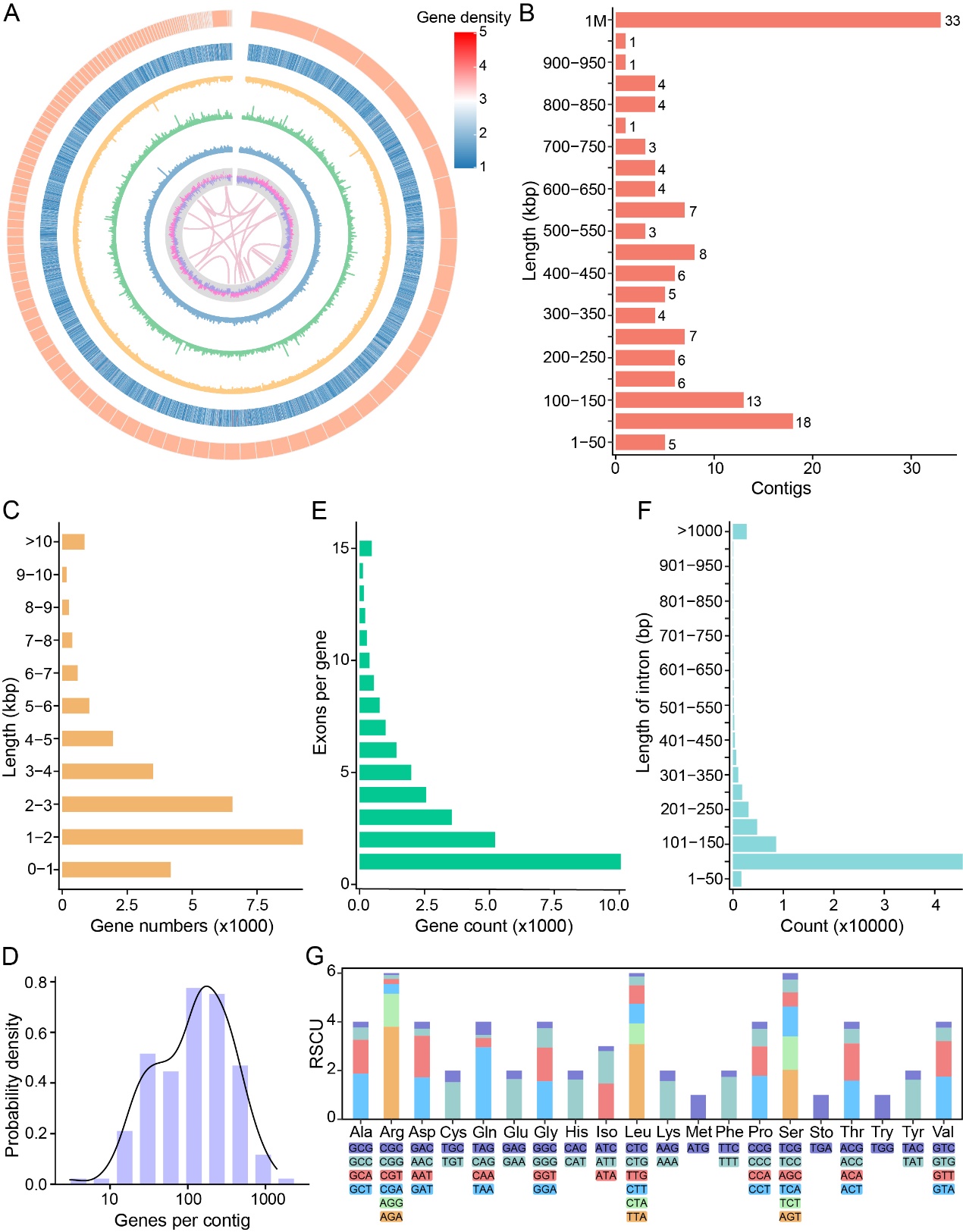
**

**Supplementary Fig. S2. Features of the *U. marinum* PJ20101A assembly.** **(A)** Circular visualization of genomic features (outermost to innermost): contig boundaries; gene density (color scale: up-right); PacBio sequencing depth; Illumina sequencing depth; GC content; GC skew. Interconnecting lines represent collinear gene blocks across contigs. The GC content and GC skew were calculated in 20 kbp sliding windows (5 kbp step size). **(B)** Distribution of contig length. **(C)** Distribution of gene length with introns. **(D)** Distribution of gene number per contig. **(E)** Distribution of exons per gene. **(F)** Distribution of intron length. **(G)** Relative synonymous codon usage (RSCU) distribution. Sto represents the stop codon.


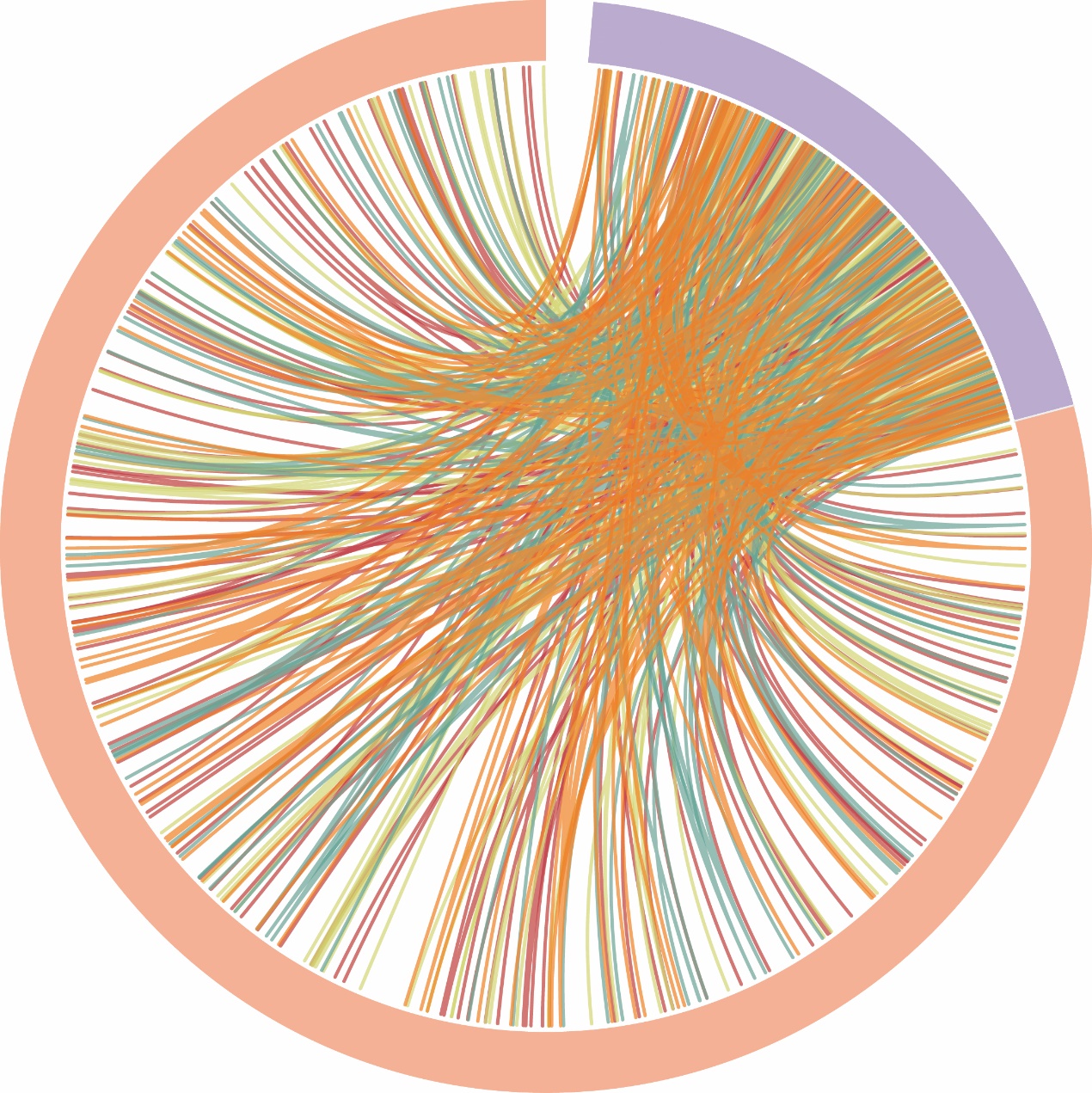


**Supplementary Fig. S3. Homologous gene pairs between *Uronema marinum* PJ20101A (orange) and *Chaenea vorax* PJ13002 (purple).** Line colors are only for reducing visual clutter.

**
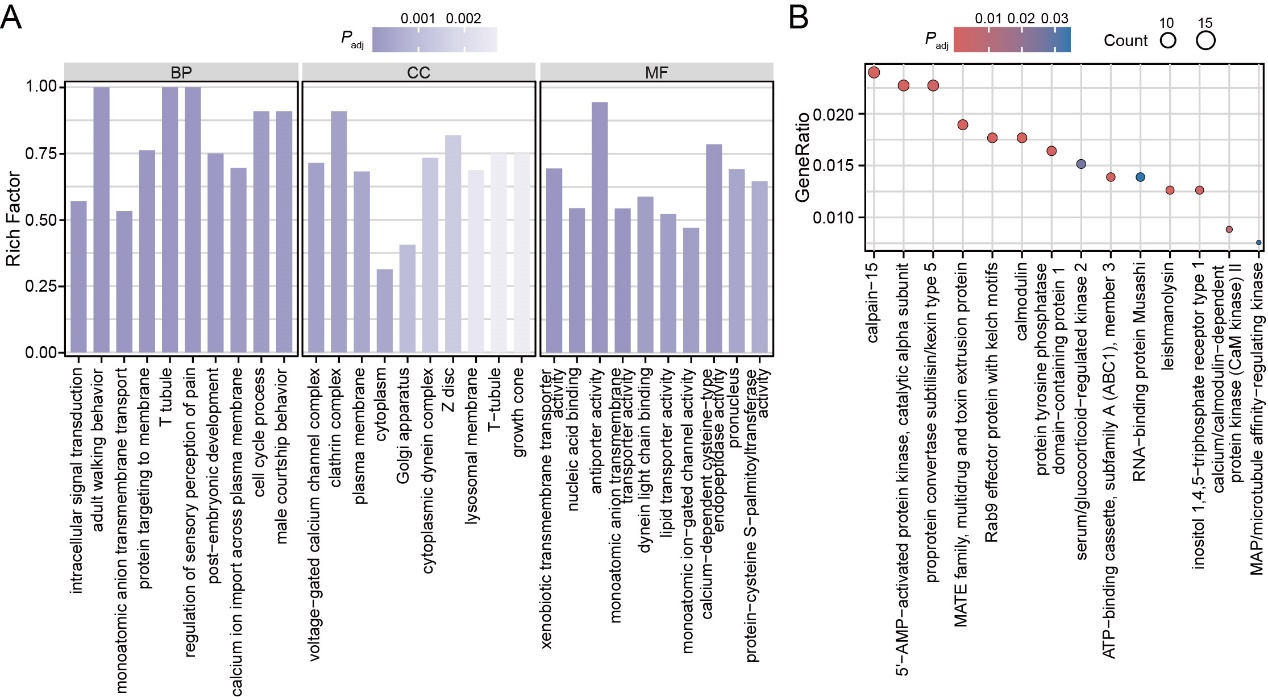
**

**Supplementary Fig. S4. Functional enrichment analysis of expanded gene families in *Chaenea vorax* PJ13002.** **(A)** Gene Ontology (GO) enrichment of the top 10 expanded gene families, categorized by Biological Process (BP), Cellular Component (CC), and Molecular Function (MF). **(B)** KEGG pathway enrichment analysis of genes from all expanded gene families.


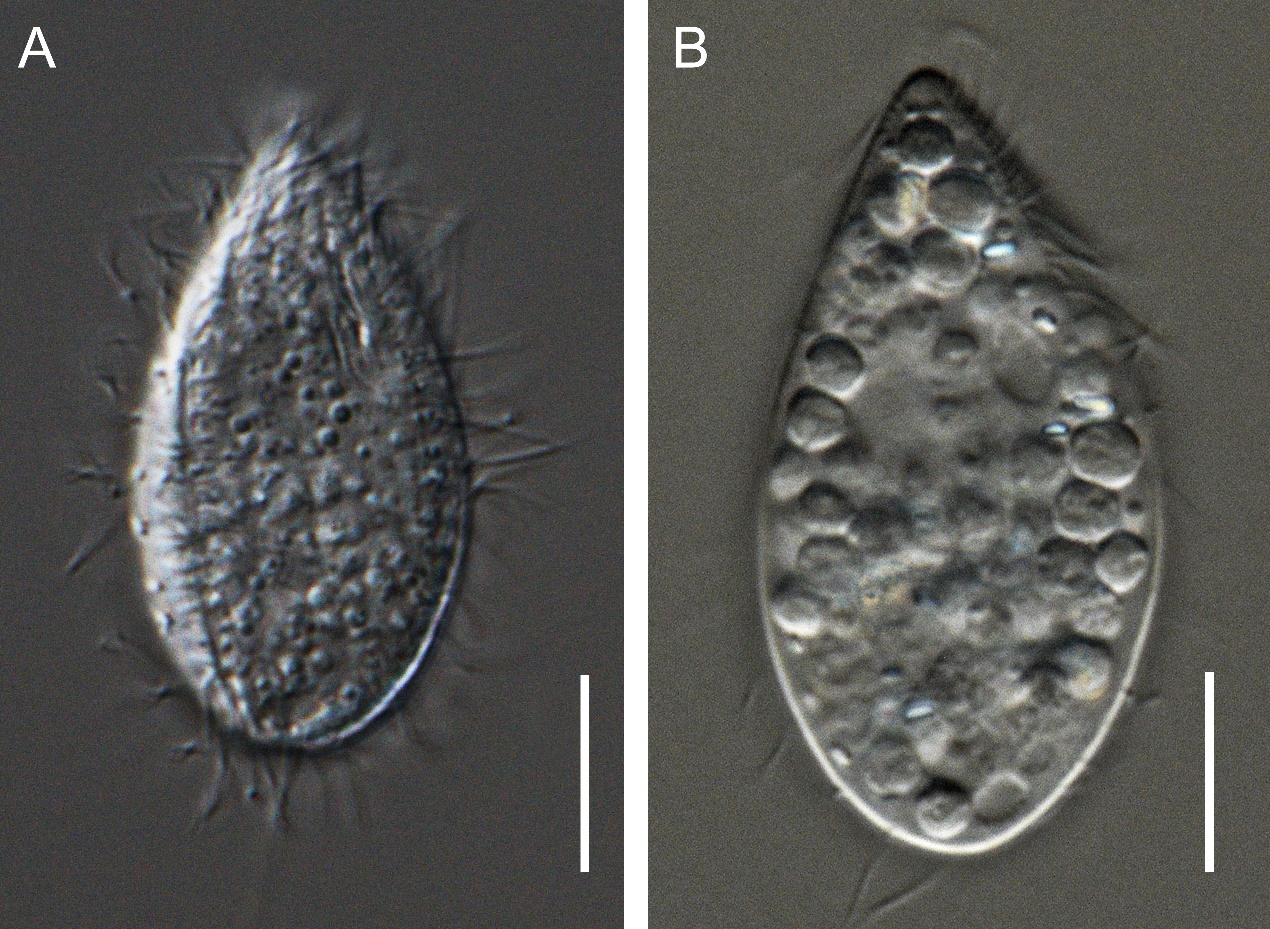


**Supplementary Fig. S5.** **Morphology of two prey ciliates of *C. vorax*. (A)** *Miamiensis avidus* PJ512A; **(B)** *Metanophrys* sp. NJH45I. Scale bars: 10 μm.


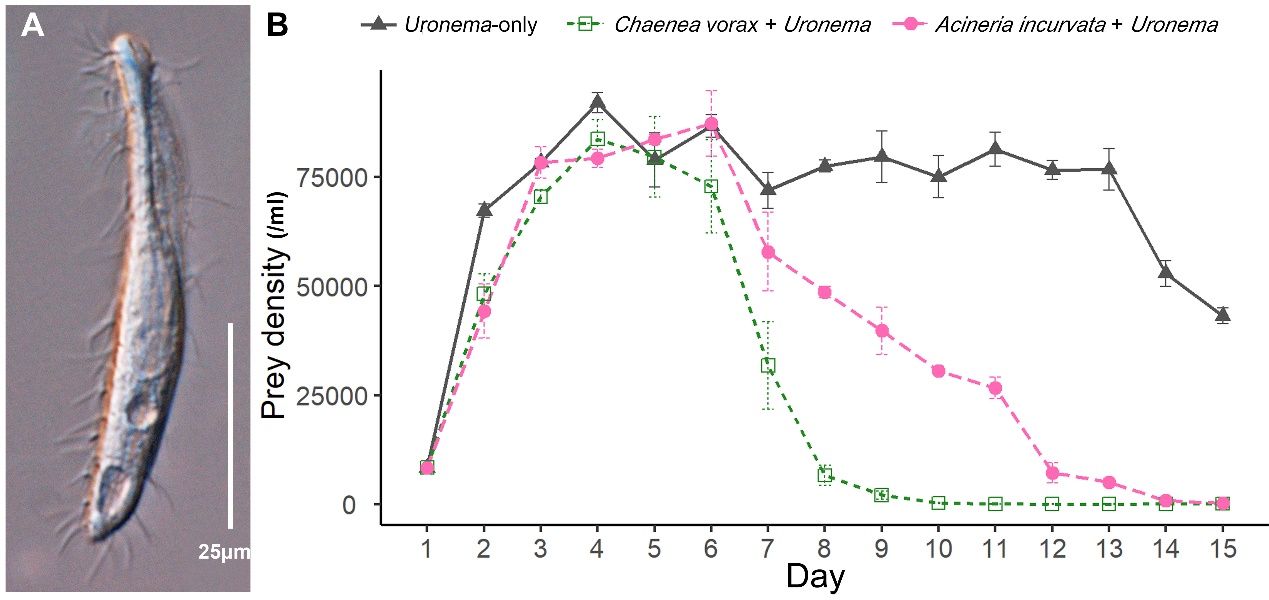


**Supplementary Fig. S6. Comparative analysis of the predator ciliate *Acineria incurvata* PJ810C and its predation efficiency on *Uronema marinum*. (A)** Morphology of the predatory ciliate *A.* *incurvata* PJ810C; **(B)** Growth dynamics of *U. marinum* under three conditions: in monoculture (control, gray triangles), in co-culture with the predator *C. vorax* (green squares), and in co-culture with the predator *A. incurvata* (pink circles).
