# Supplementary material for: Biocontrol potential and molecular basis of predation in a marine raptorial ciliate
Source: ISME J. 2026 Mar 13;20(1):wrag053. doi: 10.1093/ismejo/wrag053 (PMC13096750; doi:10.1093/ismejo/wrag053)
Supplement: wrag053_Supplemental_Files [file wrag053_supplemental_files.zip › Supplementary_Text_for_ISME_-_20260310_wrag053.docx]

**Biocontrol potential and molecular basis of predation in a marine raptorial ciliate**

Jiao Pan^1,2^, Jiahao Ni^1^, Yaohai Wang^1^, Ziguang Deng^1^, Hongwei Yue^1^, Kangqiao Dong^3^, Yichen Li^1^, Zhongze Lei^1^, Ziming Ma^1^, Gongze Hu^1^, Runda Chi^1^, Zhongyu Chang^1^, Qikai Chen^1^, Yujun Cai^1^, Hanlin Shen^1^, Runzhi Shi^1^, Wei Yang^1^, Xinpeng Fan^3^, Weiyi Li^4^, Zhiqiang Ye^5^, Michael Lynch^6^, Yu Zhang^7,*^, Hongan Long^1,2,*^

^1^ Key Laboratory of Evolution and Marine Biodiversity (Ministry of Education), Institute of Evolution and Marine Biodiversity, Ocean University of China, Qingdao, Shandong Province, China 266003

^2^ Laboratory for Marine Biology and Biotechnology, Qingdao Marine Science and Technology Center, Qingdao, Shandong Province, China 266237

^3^ School of Life Sciences, East China Normal University, Shanghai, China 200241

^4^ Department of Genetics, Stanford University School of Medicine, Stanford CA, USA 94305

^5^ School of Life Sciences, Central China Normal University, Wuhan, Hubei Province, China 430079

^6^ Biodesign Center for Mechanisms of Evolution, Arizona State University, Tempe AZ, USA 85287

^7^ School of Mathematics Science, Ocean University of China, Qingdao, Shandong Province, China 266000

**Corresponding authors**

* To whom correspondence may be addressed.

Hongan Long

[longhongan@ouc.edu.cn](mailto:longhongan@ouc.edu.cn)

Key Laboratory of Evolution and Marine Biodiversity (Ministry of Education), Institute of Evolution and Marine Biodiversity, Ocean University of China

Laboratory for Marine Biology and Biotechnology, Qingdao Marine Science and Technology Center

No. 5 Yushan Road

Qingdao, Shandong Province 266003

China

Yu Zhang

[zhangyu6929@ouc.edu.cn](mailto:zhangyu6929@ouc.edu.cn)

School of Mathematics Science, Ocean University of China

No. 238 Songling Road

Qingdao, Shandong Province 266000

China

Short title: Ciliate biocontrol potential & predation

**Supplementary Text**

**Assembly, annotation, and features of the macronuclear genome of *Uronema marinum* PJ20101A**

Using PacBio HiFi reads and Illumina PE150 short reads, we assembled a macronuclear genome of *U. marinum* PJ20101A, which has a genome size of 95.37 Mbp (Table 1; Supplementary Fig. S2). This assembly contains 143 contigs with 23 gaps, a GC content of 17%, a N50 of 1.10 Mbp, and the longest contig being 4.74 Mbp (Supplementary Fig. S2A, B; Supplementary Table S8). There are 108 contigs with at least one telomere, including 65 contigs with two telomeres (telomere sequence repeats ([C_4_A_2_]n); Supplementary Table S8). We annotated 28,748 genes for the macronuclear genome using transcriptome-based method and EuGene. The mean gene length is 2.95 kbp (Supplementary Fig. S2C, D), with an average of approximately three exons per gene (Supplementary Fig. S2E), with a mean size of 498 bp, and introns with a median size of 75 bp (Supplementary Fig. S2F). TGA is the only stop codon, while TAA and TAG are both reassigned to encode glutamine (Supplementary Fig. S2G). Additionally, we also identified 3 rRNA and 346 tRNA genes (Table 1; Supplementary Table S9). The genome also shows a strong AT bias in codon usage, matching its low genomic GC content (17%), with six AT-rich codons—AAA, TAA, AAT, TTA, TTT, and ATT—making up ~45% of all codons. GC-rich codons, particularly most arginine codons, are strongly disfavored. The mitochondrial assembly (21,456 bp) comprises 17 protein-coding genes, 2 tRNAs, and 3 rRNAs (Supplementary Fig. S1; Supplementary Table S10). It contains telomeric repeats but lacks the central AT-rich repeats typically in mitochondrial genomes of ciliates. Intragenomic collinearity analysis of *U. marinum* revealed limited collinearity, with only 118 gene pairs identified (Supplementary Fig. S2A). These represent ~0.4% of the total protein-coding genes (n = 28,748), suggesting no whole-genome duplication (WGD) in *U. marinum*. OrthoFinder analysis further showed the limited gene families in *U. marinum*, with only 879 clusters identified, consistent with the degree of lower collinearity observed. Unlike some ciliate lineages, such as certain *Paramecium* species that have undergone multiple independent WGDs leading to complex genome architectures and high paralog retention [1], the macronuclear genome of *U. marinum* seems to have been influenced predominantly by small-scale duplication events. The scarcity of large-scale duplicated blocks, along with the overall constrained gene family repertoire, suggests an evolutionary trajectory different from WGD-bearing relatives, potentially contributing to its streamlined genomic and phenotypic features.

**Functional enrichment and adaptive implications of expanded gene families in *Uronema marinum* PJ20101A**

Gene family expansions are significantly enriched in key biological processes, including translation (GO:0006412), translational elongation (GO:0006414), mRNA splicing via spliceosome (GO:0000398), and one-carbon metabolic process (GO:0006730), along with the molecular function of GTPase activity (GO:0003924). The expansion of these gene families may be due to adaptive pressures, which aim to enhance the ability to restore protein homeostasis and the coordination of metabolic-epigenetic processes. Specifically, processes related to translation and the enhancement of GTPase activity can reduce translation errors and optimize energy utilization. At the same time, the expanded splicing flexibility and methylation processes help to rapidly adapt to the environment. These mechanisms collectively support *U. marinum* being a dominant species in the marine ecosystem, and may enhance their defenses against predators.

**Reference:**

1. Ni J, Hao Y, Jiménez-Marín B, Ali F, Pan J, Wang Y, et al. Whole-genome duplications revealed by macronuclear genomes of five rare species of the model ciliates *Paramecium*. Sci China Life Sci. 2025:1–13.

**Additional information**

Additional information supporting and expanding the results presented in this study are provided as Supplementary Tables (13 display items) and Supplementary Videos (3 display items). Detailed legends are presented below.

**Supplementary Table legends**

**Supplementary Table S1.** Model comparison of alternative functional responses in the predator–prey system.

**Supplementary Table S2.** Predator and prey population densities across predation experimental groups (18°C).

**Supplementary Table S3.** Model-predicted minimum inoculum of *Chaenea vorax* required to reduce the prey population to be below 10 cells/mL within one or two days, under varying initial prey densities.

**Supplementary Table S4.** KEGG pathway enrichment analysis of all predicted genes from the *Chaenea vorax* PJ13002 *de novo* assembly. Rows highlighted with pink background indicate pathways potentially associated with the predation process.

**Supplementary Table S5.** The annotation of the mitochondrial genome of *Chaenea vorax* PJ13002.

**Supplementary Table S6.** Differentially expressed genes of *C. vorax* in predatory states vs. non-predatory states (|log_2_(FoldChange)| > =1 and *P*_adj_ <0.05). The "+" symbol in the Note indicates that the gene belongs to a gene family that has expanded.

**Supplementary Table S7.** GO and KEGG enrichment analysis of DEGs in predatory states vs. non-predatory states of *C. vorax*. 'down' or 'up' in the last column means down-regulated or up-regulated vs. the control.

**Supplementary Table S8.** Contigs with telomeres (marked with a tick), their lengths and GC content of the macronuclear genome of *Uronema marinum* PJ20101A.

**Supplementary Table S9.** Genomic locations and features of rRNA and tRNA genes in the macronulear assembly of *Uronema marinum* PJ20101A.

**Supplementary Table S10.** The annotation of the mitochondrial genome of *Uronema marinum* PJ20101A.

**Supplementary Table S11.** The genes expressed (*P*_value_ < 0.05) in U. marinum during predation by *C. vorax*. Gene expression was quantified by mapping RNAseq reads of *C. vorax* predation experiments (predation vs. non-predation) onto the *U. marinum* genome. *P*_value_ were calculated using DESeq2.

**Supplementary Table S12.** The results of GO/KEGG enrichment analysis on the genes in expanded gene families of *Chaenea vorax* PJ13002.

**Supplementary Table S13.** Strain information of the two ciliates.

**Supplementary Video legends**

**Supplementary Video 1.** Predation of *Uronema marinum* PJ20101A by *Chaenea vorax* PJ13002. Microscopy showing the predatory behavior of *C. vorax* (larger cell) attacking and consuming *U. marinum* (smaller cells). Recorded under inverted microscopy.

**Supplementary Video 2.** Predation of *Miamiensis avidus* PJ512A by *Chaenea vorax* PJ13002, featuring the morphology of *M. avidus* PJ512A (upper left). Recorded under inverted microscopy.

**Supplementary Video 3.** Predation of *Metanophrys* sp. NJH45I by *Chaenea vorax* PJ13002, featuring the morphology of *Metanophrys* sp. (upper right). Recorded under inverted microscopy.
